# Multiscale Structural Modulation of Anisotropic Graphene Framework for Polymer Composites Achieving Highly Efficient Thermal Energy Management

**DOI:** 10.1002/advs.202003734

**Published:** 2021-02-19

**Authors:** Wen Dai, Le Lv, Tengfei Ma, Xiangze Wang, Junfeng Ying, Qingwei Yan, Xue Tan, Jingyao Gao, Chen Xue, Jinhong Yu, Yagang Yao, Qiuping Wei, Rong Sun, Yan Wang, Te‐Huan Liu, Tao Chen, Rong Xiang, Nan Jiang, Qunji Xue, Ching‐Ping Wong, Shigeo Maruyama, Cheng‐Te Lin

**Affiliations:** ^1^ Key Laboratory of Marine Materials and Related Technologies Zhejiang Key Laboratory of Marine Materials and Protective Technologies Ningbo Institute of Materials Technology and Engineering (NIMTE) Chinese Academy of Sciences Ningbo 315201 P. R. China; ^2^ Center of Materials Science and Optoelectronics Engineering University of Chinese Academy of Sciences Beijing 100049 P. R. China; ^3^ Department of Mechanical Engineering University of Nevada Reno NV 89557 USA; ^4^ School of Energy and Power Engineering Huazhong University of Science and Technology Wuhan 430074 China; ^5^ National Laboratory of Solid State Microstructures College of Engineering and Applied Sciences Jiangsu Key Laboratory of Artificial Functional Materials and Collaborative Innovation Center of Advanced Microstructures Nanjing University Nanjing 210093 China; ^6^ School of Materials Science and Engineering Central South University Changsha 410083 P. R. China; ^7^ Shenzhen Institutes of Advanced Technology Chinese Academy of Sciences Shenzhen 518055 China; ^8^ Department of Mechanical Engineering The University of Tokyo 7‐3‐1 Hongo, Bunkyo‐ku Tokyo 113‐8656 Japan; ^9^ Energy Nano Engineering Laboratory National Institute of Advanced Industrial Science and Technology (AIST) Tsukuba 305‐8564 Japan; ^10^ School of Materials Science and Engineering Georgia Institute of Technology Atlanta GA 30332 USA

**Keywords:** junction thermal resistance, multiscale structural modulation, phase change composite, thermal interface material, vertically aligned graphene

## Abstract

Graphene is usually embedded into polymer matrices for the development of thermally conductive composites, preferably forming an interconnected and anisotropic framework. Currently, the directional self‐assembly of exfoliated graphene sheets is demonstrated to be the most effective way to synthesize anisotropic graphene frameworks. However, achieving a thermal conductivity enhancement (TCE) over 1500% with per 1 vol% graphene content in polymer matrices remains challenging, due to the high junction thermal resistance between the adjacent graphene sheets within the self‐assembled graphene framework. Here, a multiscale structural modulation strategy for obtaining highly ordered structure of graphene framework and simultaneously reducing the junction thermal resistance is demonstrated. The resultant anisotropic framework contributes to the polymer composites with a record‐high thermal conductivity of 56.8–62.4 W m^−1^ K^−1^ at the graphene loading of ≈13.3 vol%, giving an ultrahigh TCE per 1 vol% graphene over 2400%. Furthermore, thermal energy management applications of the composites as phase change materials for solar‐thermal energy conversion and as thermal interface materials for electronic device cooling are demonstrated. The finding provides valuable guidance for designing high‐performance thermally conductive composites and raises their possibility for practical use in thermal energy storage and thermal management of electronics.

## Graphical abstract

The dual‐assembled graphene framework (DAGF), which combines a highly oriented graphene structure along the vertical direction and a low junction thermal resistance between the graphene building blocks, exhibits excellent performance in improving the thermal conductivity of polymers. The resultant composites have a record‐high thermal conductivity of 56.8–62.4 W m^−1^ K^−1^(≈13.3 vol%), significantly outperforming previously reported results.

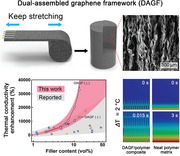

## 1 Introduction

In recent decades, along with the rapid development of electronic and energy technologies, a serious issue concerning thermal energy management has gradually emerged and is becoming of crucial importance for improving the performance of various devices.^[^ ^1^, ^2^, ^3^ ^]^ For example, in semiconductor industries, the shrinking feature size and escalating power density of transistors and integrated circuit packaging promote a significant enhancement of the computing capability, meanwhile resulting in a great increase of heat dissipation across the chip, board, and system levels.^[^ ^4^, ^5^ ^]^ The accompanying interfacial heat transfer problem leads to an urgent demand for thermal interface materials (TIMs) with high thermal conductivity for removing excess thermal energy to guarantee the continuous and stable operation of the electronic devices.^[^ ^6^, ^7^, ^8^ ^]^ And in the field of thermal energy harvesting based on the phase‐change technology, the low intrinsic thermal conductivity (<0.5 W m^−1^ K^−1^) of the phase‐change materials (PCMs) is a long‐standing bottleneck, which greatly limited the thermal charging/discharging rate, thus causing a low heat‐utilization efficiency for diverse applications of PCMs, such as solar‐thermal energy conversion, thermal management of batteries, and thermal diodes.^[^ ^9^, ^10^ ^]^ Therefore, addressing the thermal energy management issue by developing advanced thermally conductive materials has become necessary for the sustainable and stable development of the electronics and energy industries.

Graphene is a monolayer of covalently sp^2^‐hybridized carbon atoms in a honeycomb lattice, exhibiting an extremely high thermal conductivity over 5000 W m^−1^ K^−1^.^[^ ^11^, ^12^, ^13^ ^]^ Such an excellent heat conduction performance has triggered considerable research interest in developing diverse graphene‐based materials to meet the ever‐increasing thermal energy management requirement, such as graphene papers as heat spreaders, graphene aerogels for solar thermal generation, and graphene/polymer composites.^[^ ^14^, ^15^, ^16^, ^17^, ^18^ ^]^ In particular, graphene/polymer composites, which were prepared by embedding graphene into polymer matrices, have been continuously spotlighted and implemented various applications in the electronics and energy field, due to the improved heat conductance, low density, and ease of processing.^[^ ^19^, ^20^, ^21^ ^]^ Currently, the direct dispersion of chemically exfoliated graphene sheets in polymer matrices by a solution or melt‐blending process is the most common way to prepare the composites.^[^ ^22^, ^23^ ^]^ In this case, the critical issue is the high intrinsic interfacial thermal resistance between dispersed graphene sheets and polymer matrices, greatly limiting the thermal conductivity enhancement of the resultant composites. ^[^ ^17^, ^24^ ^]^ Generally, to achieve an efficient thermal percolation pathway, a high graphene content up to 20–30 vol% in the polymer matrix is required, but nonetheless, the currently reported thermal conductivity enhancement (*TCE =* (*κ − κ*_m_)/*κ*_m_ × 100%) is mostly lower than 3000%, resulting in a poor thermal conductivity of 4–6 W m^−1 ^K^−1^,^[^ ^20^, ^25^ ^]^ where *κ* and *κ*_m_ are the thermal conductivity of composites and polymer matrix, respectively. Accordingly, we can obtained that the specific *TCE* (*TCE* per 1 vol% filler addition) of the polymer matrices using the dispersed graphene as filler is almost less than 200%, inherently restricting the real thermally conductive applications of the corresponding composites.^[^ ^23^, ^26^, ^27^, ^28^ ^]^

Recently, 3D graphene frameworks composed of interconnected graphene sheets have emerged as ideal reinforcements to develop thermally conductive polymer composites, due to the formation of the continuous thermal pathway between graphene fillers for rapid phonon transport.^[^ ^29^, ^30^, ^31^ ^]^ And more effective thermal conductivity enhancement can be achieved by modulating the graphene framework to form a highly ordered and anisotropic structure instead of random arrangement.^[^ ^16^, ^32^ ^]^ It can be attributed to the fact that the thermal conductivity of graphene is highly anisotropic, having an excellent capability to transfer heat along the basal plane, but poor along its cross‐plane direction.^[^ ^33^, ^34^ ^]^ Current methods for the development of anisotropic graphene frameworks using graphene sheets, such as graphene oxide (GO), reduced GO (rGO), or graphite nanoplatelets (GNPs), can be typically divided into two approaches: the directional‐freezing of rGO aqueous dispersion and self‐assembly of GO liquid crystals.^[^ ^35^, ^36^, ^37^, ^38^ ^]^ The former was usually carried out based on an ice‐templated assembly strategy, by which the graphene sheets can be spontaneously restacked on the edges of the unidirectional grown ice crystals to form an ordered arrangement.^[^ ^39^, ^40^, ^41^ ^]^After incorporating with polymer matrices, typically, the thermal conductivity of the composites along the preferred direction can be up to 2.13 W m^−1^ K^−1^ with a graphene content of 0.92 vol%, corresponding to the specific TCE of 1338%.^[^ ^19^ ^]^ The anisotropic graphene framework can be also readily prepared utilizing the liquid crystal nature of GO, which arises from their intrinsic shape anisotropy and mutual electrostatic‐repulsion, leading to the spontaneous assembly of GO with a long‐range ordered structure in the aqueous dispersion.^[^ ^42^, ^43^, ^44^ ^]^ After air‐drying followed by a graphitization treatment at 2800 °C, Yu et al. embedded the as‐prepared anisotropic graphene framework into the epoxy, and obtained a currently record‐high thermal conductivity of 35.5 W m^−1^ K^−1^ for the graphene/polymer composites (19 vol%), giving a specific TCE up to 884% along the preferred direction.^[^ ^45^ ^]^ Although the above strategies have achieved a significant thermal conductivity improvement for polymers by incorporating the anisotropic graphene frameworks into the matrices, the thermal resistance between the adjacent graphene sheets within those frameworks is still fairly high. It can be attributed to that the graphene frameworks prepared through the commonly used spontaneous assembly method could just form an incompact contact between the adjacent graphene sheets with a low overlapping area of graphene‐graphene.^[^ ^20^ ^]^ Such an interfacial thermal resistance at the microscopic junction can ultimately contribute to the high total thermal resistance inside bulk composites, limiting the specific TCE mostly below about 1500% for the anisotropic graphene framework/polymer composites ever reported.^[^ ^9^, ^19^, ^45^, ^46^, ^47^ ^]^ Therefore, further interface optimization for reducing the junction thermal resistance between the adjacent graphene sheets within the anisotropic graphene framework is imperative to further improve the thermal conductivity of graphene‐based polymer composites.

Herein, to address this issue, we proposed a dual‐assembly strategy with a multiscale structural modulation process to construct anisotropic graphene framework, which has not only a highly oriented arrangement of graphene along the vertical direction, but also an intimate contact of the adjacent graphene sheets with a low junction thermal resistance. As a result, the dual‐assembled graphene framework (DAGF) exhibited an excellent thermal conductivity enhancement effect on various polymer matrices, typically endowing the epoxy composites with a through‐plane thermal conductivity of 62.4 W m^−1^ K^−1^ (≈13.3 vol%). This value achieved is equivalent to ≈325 times higher than that of neat epoxy, giving an ultrahigh specific TCE over 2400%. Additionally, given practical applications of thermal energy management, we further incorporated the as‐prepared DAGF with the polyethylene glycol (PEG) and the polydimethylsiloxane (PDMS), and the both demonstrated a superior performance using as PCMs, and TIMs, respectively. Our finding provides insight into the construction of graphene‐based thermally conductive composites, which may meet the ever‐increasing thermal energy management issue in electronics and energy fields.

## 2 Results and Discussion

### 2.1 Preparation and Structural Characterization of DAGF

**Figure 1:**
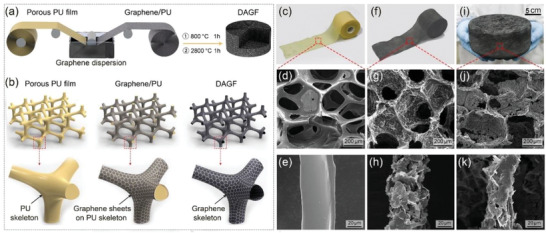
Schematic illustrating the a) fabrication process of the DAGF and b) the corresponding structural change of each step based on the proposed dual‐assembly method. Optical and SEM images of the c–e) porous PU film, f–h) graphene/PU, and i–k) DAGF, respectively.

**Figure** **1**a illustrates our strategy for fabricating the DAGF with the corresponding structural change of each step during the preparation process showing in Figure 1b. The detailed description of the whole procedure can be found in the Experimental Section. In brief, the graphene/polyurethane (graphene/PU) was prepared first through a dual‐assembly method, which adopted a porous PU thin film as the starting template to assemble graphene sheets on PU skeletons using solution immersion process, followed by a continuous roll‐to‐roll step to postassemble the film into a large scale monolith using a self‐developed roller equipment. As shown in Figure 1c,d, commercial PU film with the thickness of ≈500 µm has a continuous and interconnected macropore structure, whose cellular size is in the range of 200–400 µm, and in Figure 1e, the internal skeleton of the PU film presents a fairly smooth surface with a wire diameter of ≈40 µm. After the continuous immersion of the PU film into the graphene/ethanol dispersion (10 mg mL^−1^), the color of the film turned from yellow to black (Figure 1b,f), due to the uniform coating of graphene sheets onto the PU skeleton (Figure 1g). In Figure 1h, it can be observed that graphene sheets preferred to be face‐to‐face attached on the surface of the PU skeleton, due to the ultrathin nature of graphene sheets with a high aspect ratio (> 500) and the relatively strong adsorption interaction between graphene sheets and the PU based on van der Waals (vdW) forces.^[^ ^48^, ^49^ ^]^ In the subsequent roll‐to‐roll process, the black film was continuously rolled up into a cylindrical monolith (graphene/PU), and the sample size was mainly depended on the length of the film used in the process. For example, a large‐scaled graphene/PU with a diameter of ≈20 cm and a thickness of ≈8 cm can be obtained by continuously rolling up a 60 m long film. Finally, as shown in Figure 1i, the DAGF with the same dimension was synthesized by the pyrolysis of graphene/PU at 800 °C (1 h) to remove the PU template. And the quality of DAGF can be further improved by post‐thermal annealing at 2800 °C (1 h) for repairing the structural defects and enlarging the domain size of the graphene, based on the Raman and XRD (X‐ray diffraction) analyses shown in Figure S1 (Supporting Information). Figure 1j indicates that the resultant DAGF can still maintain the characteristic interconnected cellular structure with a slight contraction of the skeleton (Figure 1k) compared to the original morphology of the PU film.

**Figure 2:**
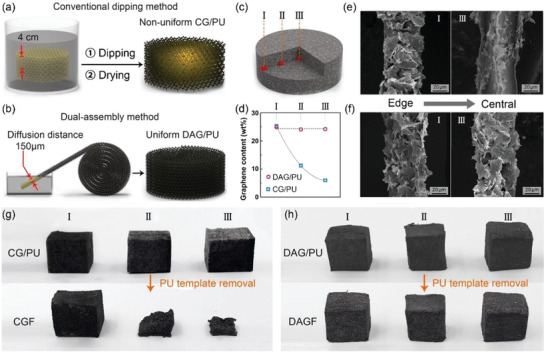
Schematic illustrating the incorporation of graphene sheets and the porous PU sponge using a) conventional dipping method and b) our proposed dual‐assembly method to fabricate the graphene/PU. b) Schematic illustrating three different sampling regions from edge to the central within graphene/PU. c) The graphene content in the different regions of the two types of graphene/PU, with the corresponding SEM images showing in (e) and (f) for the case of CG/PU and DAG/PU, respectively. The photograph of selected graphene/PU cubies before and after the PU template removal for the case of e) CG/PU and f) DAG/PU.

Actually, the fabrication of interconnected graphene frameworks using porous PU sponge as the sacrifice template has been reported in previous works.^[^ ^50^, ^51^, ^52^ ^]^ And the commonly used method was performed according to a “dipping and drying” process illustrated in **Figure** **2**a, in which the bulk PU sponge was directly immersed into the graphene dispersion for coating graphene sheets on its skeleton, followed by thermal annealing for the removal of the PU template to obtain the graphene framework.^[^ ^53^ ^]^ However, it is difficult for conventional dipping method to achieve a large scale graphene framework while guaranteeing the homogeneity of the entire structure. It is mainly because when a large‐scale PU sponge was simply immersed into the graphene dispersion, the graphene sheets attached at the outer surface of the sponge would form a diffusion barrier layer to prevent the continuous penetration of dispersion toward the central region, finally leading to the nonuniform assembly of graphene sheets after drying. In contrast, the dual‐assembly strategy proposed in this study adopted the porous PU film with the thickness of 500 µm (Figure S2, Supporting Information) as the starting template to immerse into the graphene dispersion, by which graphene sheets can diffuse from the normal direction of the film with a short diffusion distance of 250 µm. As illustrated in Figure 2a,b, when preparing a same size graphene framework shown in Figure 1i (Φ 20 × 8 cm^3^), the diffusion distance of our dual‐assembly method (250 µm) is almost 16 times shorter than that of the conventional dipping method (4 cm), leading to the ease of uniformly attaching the graphene sheets on entire surface of the PU skeletons. The subsequent roll‐to‐roll step was carried out to postassemble the as‐prepared homogeneous graphene/PU film into a large‐scale graphene/PU monolith with uniformly distributed graphene sheets.

In order to intuitively demonstrate this point, the microscopic morphologies and graphene content at different regions from the edge to the center of the dual‐assembled graphene/PU (DAG/PU) monolith (Φ 20 × 8 cm^3^) was investigated, with the three typical sampling regions illustrated in Figure 2c. For comparison, a control experiment was carried out on the same scale of conventional graphene/PU (CG/PU) prepared using the common “dipping and drying” method. Based on the thermogravimetric analysis (Figure 2d; and Figure S3, Supporting Information) and acquired scanning electron microscopy (SEM) images (Figure 2e), three sampling regions of DAG/PU exhibit a similar morphology with an approximate content of graphene sheets attaching on the PU skeleton surface, confirming the uniform distribution of graphene sheets within DAG/PU. In sharp contrast, an apparent diminishing in the graphene content can be found from the edge to the central region for the CG/PU case (Figure 2d,f; and Figure S3, Supporting Information). As a result, based on the distribution difference of graphene sheets within the two types of graphene/PU, the annealing of three graphene/PU cubes (2 × 2 × 2 cm^3^) cut out from the three sampling regions in DAG/PU and CG/PU exhibits fundamentally different results. In Figure 2g,h, DAGFs present nearly consistent shape compared to the original graphene/PU cubes, whereas the structural collapse of CGFs (conventional graphene frameworks) can be found for the sampling regions away from the edge of CG/PU monolith after the template removal, due to the lack of enough graphene sheets to support the structure. This result indicates that our proposed dual‐assembly strategy is more feasible compared to the commonly used “dipping and drying” method for the construction of nanosized graphene sheets into a homogeneous graphene framework with a large scale.

**Figure 3:**
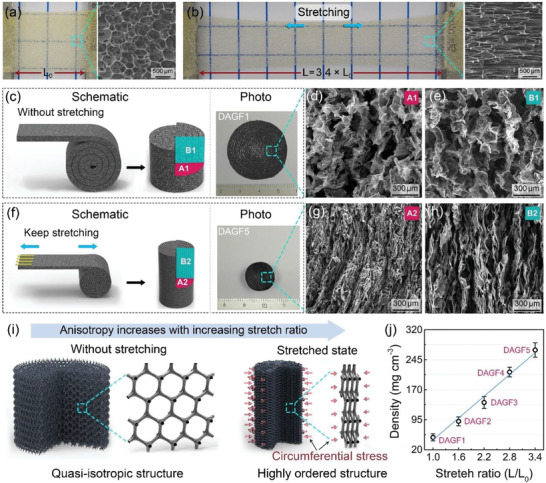
The photographs and the corresponding top‐view SEM images of a) unstretched and b) 3.4‐fold stretched porous PU film. c) Schematic, photograph, d) top‐view, and e) cross‐sectional SEM images of DAGF prepared using the unstretched PU porous film (DAGF1). f) Schematic, photograph, g) top‐view, and h) cross‐sectional SEM images of DAGF prepared through controlling the film stretch ratio of 340% (DAGF5). i) Schematic illustrating the modulation of DAGF structure from quasi‐isotropic to highly ordered arrangement with the increase of stretch ratio. j) The densities of the resultant DAGFs as a function of stretch ratio.

The proposed dual‐assembly strategy can not only efficiently construct a homogeneous graphene framework with a large scale, but also manipulate the structural orientation of the resultant framework, based on the stretchable nature of the porous PU film. As shown in **Figure** **3**a,b, the PU film can be easily stretched out to ≈3.4 times compared to its original length, with the isotropic pores elongating into spindle shape for the stretched sample. According to this feature, a series of DAGFs (DAGF1–DAGF5) can be prepared by controlling the PU film during the roll‐to‐roll process with different stretch ratio (*L*/*L*_0_ = 1, 1.6, 2.2, 2.8, 3.4), where *L* and *L*_0_ are the length of the pristine and the stretched PU porous film, respectively. The detailed preparation process of the DAGFs can be found in Figure S4 (Supporting Information), and the microstructure change of them were presented in Figure 3c–h. As shown in Figure 3c, the DAGF prepared using the unstretched PU porous film (*L*/*L*_0_ = 1) as the starting template (named as DAGF1) exhibits a fairly loose skeleton structure composed of quasi‐isotropic arrangement of graphene sheets (Figure 3d,e). Based on this characteristic structure, the roll‐up of a 1.6 m long film can finally achieve a cylindrical graphene framework with a diameter of ≈32 mm (Figure 3c). In contrast, by rolling‐up a 3.4‐fold stretched film (*L*/*L*_0_ = 3.4) with the same original length, the resultant DAGF (DAGF5) presents an obviously lessened diameter of ≈15 mm (Figure 3f). Interestingly, as comparison with the loose and quasi‐isotropic porous structure of DAGF1 (Figure 3d,e), the DAGF5 has a densely packed structure composed of highly ordered arrangement of graphene sheets toward the vertical direction, as shown in Figure 3g,h. We proposed that the microstructure change of the DAGFs from quasi‐isotropic to anisotropic with an increase of the PU film stretch ratio can be attributed to the excellent stretchability of the applied film. In the continuous roll‐to‐roll step, when the elastic film under stretched state was rolled up into a cylindrical shape, the natural contraction behavior of the PU film provides a constant circumferential stress along the normal direction of the film, as demonstrated in Figure S5 (Supporting Information). The generated circumferential stress can drive the quasi‐isotropic graphene skeleton to transform into a highly ordered structure composed of vertically aligned graphene sheets (Figure 3i). In addition, the density of the resultant DAGFs also increases with an increase of PU film stretch ratio, as the results shown in Figure 3j, in which the highest density of ≈270 mg cm^−3^ for the DAGF5 can be achieved at a stretch ratio of 340%. Note that the density increase versus the stretch ratio was nearly linear, indicating a good controllability for modulating the density and the structural orientation of the graphene framework using our proposed dual‐assembly strategy.

### 2.2 Thermal Conductivity of DAGF/Polymer Composites

**Figure 4:**
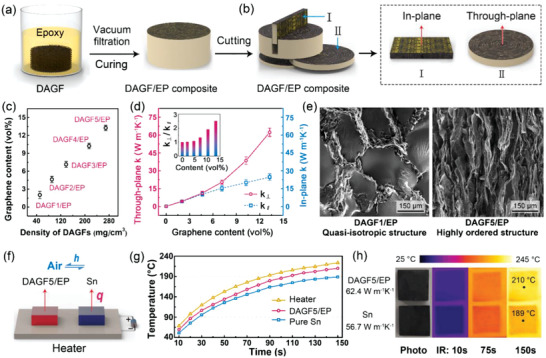
Scheme illustrating a) the fabrication process of the DAGF/EP composites, and b) the cutting of the sample into small pieces for the detection of thermal conductivities along in‐plane and through‐plane direction. c) The volume fraction of graphene in the DAGF/EP composites versus the density of the DAGFs. d) The in‐plane (*κ*_∥_) and through‐plane (*κ*_⊥_) thermal conductivities of DAGF/EPs as a function of graphene content and the inset presenting the thermal conductivity anisotropy ratio (*κ*_⊥_/*κ*_∥_). e) The cross‐sectional SEM images of DAGF1/EP and DAGF5/EP. f) The test system configuration for demonstrating the through‐plane heat transfer capacity. g) Surface temperature evolution and h) the corresponding IR images of DAGF5/EP and Sn versus heating time.

Based on the characteristic interconnected structure composed of highly ordered graphene sheets, the DAGF is expected to be a promising candidate as thermally conductive fillers embedded into polymer matrices to develop composites with improved thermal conductivity for highly efficient thermal energy management. To confirm this, the DAGF/epoxy (EP) composites were prepared, and the contribution of the DAGFs on the heat transfer capability of epoxy was studied. Epoxy was chosen because it is not only a widely utilized thermal management material in the electronic packaging field, but also the most commonly used polymer matrix for evaluating the heat transfer enhancing effect of the applied fillers in academia. In **Figure** **4**a, a vacuum infiltration of epoxy, followed by thermal curing was adopted to prepare the DAGF/EP composites, which were further cut into small pieces for the determation of their thermal conductivity along in‐plane and through‐plane direction (Figure 4b) using laser flash technique. The detailed measurement process of the thermal conductivity using laser flash technique can be found in Section S1 (Supporting Information). According to the five types of DAGFs with varying densities (Figure 3j), a group of DAGF/EPs (named as DAGF1/EP–DAGF5/EP) could be obtained, as shown in Figure 4c, in which the volume fraction of graphene in the DAGF/EPs was determined based on thermogravimetric analysis (TGA) analysis (Figure S6, Supporting Information). In Figure 4d and Table S1 (Supporting Information), the composite with the lowest graphene content (DAGF1/EP, ≈2 vol%) exhibits in‐plane (*κ*_∥_) and through‐plane (*κ*_⊥_) thermal conductivities of 3.98 and 4.07 W m^−1 ^K^−1^, respectively, indicating an approximately isotropic heat transfer enhancing effect of DAGF1. When the DAGFs with higher density were incorporated, both *κ*_∥_ and *κ*_⊥_ of DAGF/EPs present a significant improvement as a function of graphene content. Interestingly, we noticed that the *κ*_∥_ rises in an almost linear trend, whereas the increase of *κ*_⊥_ is approximately exponential, leading to a monotonical increase of the thermal conductivity anisotropy ratio (*κ*_⊥_/*κ*_∥_) versus the graphene content (the inset of Figure 4d). As a result, by combining the highest‐density DAGF with epoxy, the *κ*_∥_ and *κ*_⊥_ of composite (DAGF5/EP, ≈13.3 vol%) can reach 24.8 and 62.4 W m^−1 ^K^−1^, respectively, corresponding to a thermal conductivity anisotropy ratio (*κ*_⊥_/*κ*_∥_) of 2.52. The changing trend from approximately isotropic to anisotropic of thermal conductivity enhancement effect for the DAGFs on the epoxy as the increase of graphene content can be attributed to the structural change of the framework as discussed in Figure 3. The comparison between Figures 3e,h and 4e indicates that, after incorporating with the epoxy, the DAGFs can maintain the characteristic structure within the matrix, based on the moderate infiltration process and good structural stability of the framework (Figure S7, Supporting Information). As expected, the DAGF1/EP was embedded by a quasi‐isotropic arrangement of the graphene skeleton, which endows the composite with an approximately isotropic thermal conductivity. In contrast, by incorporating the DAGF5 with a highly anisotropic structure, as elaborated in Figure S8 (Supporting Information), the corresponding epoxy composite showed a preference for heat conduction along the through‐plane direction. Particularly, based on the formation of efficient heat pathways toward the vertical direction composed of highly ordered graphene, the *κ*_⊥_ achieved of DAGF5/EP (62.4 W m^−1 ^K^−1^) is over two orders‐of‐magnitude greater than that of polymer (0.2–0.4 W m^−1 ^K^−1^), and outperforms that of many metals and ceramics.^[^ ^54^ ^]^

Furthermore, we carried out a comparative test on through‐plane heat transfer capacity between DAGF5/EP and tin (Sn) for directly demonstrating the ultrahigh *κ*_⊥_ of our composite. In Figure 4f, DAGF5/EP and Sn (≈56.7 W mK) with the same size of 10 × 10 × 3 mm^3^ were placed on a ceramic heater (60 W) for heating them at the same time from room temperature. To precisely measure the time‐dependent surface temperature of different materials using a commercial infrared (IR) camera, the top surface of the two samples was coated by a thin graphite layer (≈5 µm) to ensure the same infrared emittance. As the results shown in Figure 4g,h, when the test started, the surface temperature of DAGF5/EP rises faster compared to that of Sn, leading to a significant temperature difference of 21 °C at 150 s, honestly determining the metal‐level heat transfer capacity of DAGF5/EP along the through‐plane direction.

**Figure 5:**
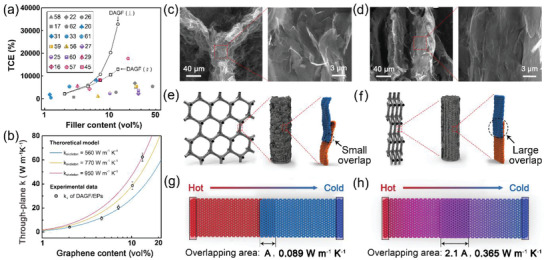
a) Comparison of thermal conductivity enhancement (TCE) of our DAGF/EP composites with reported graphene/polymer composites. b) Fitting of the experimental *κ*_⊥_ of DAGF/EP composites based on the foam theory. c,d) The morphologies and e,f) scheme illustrating the rearrangement of DAGF including the graphene skeleton and the graphene sheets during the dual‐assembly process. c,e) and d,f) show the cases of low‐density DAGF (DAGF1) and high‐density DAGF (DAGF5), respectively. The calculated junction thermal conductance of adjacent graphene sheets with g) small and h) large overlapping area based on the NEMD simulation. The arrow shows the direction of the heat flux.

In order to comprehensively evaluate the heat transfer enhancing effect of our DAGFs on the polymer matrix, a comparison of TCE between our DAGF/EPs and reported graphene/polymer composites was exhibited in **Figure** **5**a; and Table S2 (Supporting Information).^[^ ^16^, ^17^, ^20^, ^22^, ^25^, ^26^, ^27^, ^29^, ^31^, ^45^, ^55^, ^56^, ^57^, ^58^, ^59^, ^60^, ^61^, ^62^ ^]^ The *κ*_∥_ enhancement of DAGF/EPs presents a relatively consistent rising pattern as compared to the general trends of the currently reported results. However, note that the TCE of *κ*_⊥_ shows an accelerated growth rate versus the graphene content, and begins to significantly outperform that of the latest reports when the graphene content exceeds about 10 vol%. To understand such an extraordinary *κ*_⊥_ enhancement of DAGF/EPs, our experimental *κ*_⊥_ was matched using a heat conduction model according to the metal foam theory, which takes the foam skeleton as the research object, and combines the *κ*_skeleton_ and the *κ*_EP_ through a rule of mixtures^[^ ^16^, ^20^, ^31^ ^]^

(1) $$\kappa_{⊥}=\langle \cos^{2}\theta \rangle f\kappa_{\mathrm{skeleton}}+\left(1-f\right)\kappa_{\mathrm{EP}}$$

where *κ*_EP_ is the thermal conductivity of the epoxy matrix; *κ*_skeleton_ is the solid thermal conductivity of an individual graphene skeleton of DAGFs; *f* is the volume fraction of graphene; *θ* is the angle between the graphene skeleton and the direction of heat transfer, and the angle bracket indicates the average value over all graphene skeleton. The detailed calculation and analysis can be seen in Section S2 (Supporting Information). As the results shown in Figure 5b, when the value of *κ*_skeleton_ was taken as 560 W m^−1^ K^−1^, the predicted results of Equation (1) can well fit the first three points (*f* < 7.2 vol%) of our experimental data, whereas underestimating the *κ*_⊥_ of the last two points, which can be matched by assigning a higher *κ*_skeleton_ of 770 and 950 W m^−1^ K^−1^ for Equation (1), respectively. Based on the correlation between the graphene content in DAGF/EP composites and the density of the applied DAGFs (Figure 4c), it is reasonable to assume that there should exist another mechanism, which contributes to the higher‐density DAGF having a superior heat conduction capability of the individual graphene skeleton.

Figure 5c,d shows the comparative morphologies of the graphene skeleton within the DAGF1 (the lowest density) and DAGF5 (the highest density), respectively. It is obvious that, different from the rough skeleton composed of the loosely stacked graphene sheets for DAGF1, the skeleton surface of DAGF5 is fairly smooth with a dense stacking of graphene sheets, indicating a highly ordered arrangement along the direction of the skeleton. Accordingly, we proposed that the dual‐assembly strategy with a continuous stress‐induced orientation process can not only manipulate the arrangement of the graphene skeleton, leading to the formation of highly ordered structure, but also further optimize the stacking order of the graphene sheets with the increased overlapping area, as schematically illustrated in Figure 5e,f. This effect is similar to the rearrangement of graphene sheets within the graphene paper toward the horizontal direction by applying a vertical compression.^[^ ^63^ ^]^ Based on the construction of anisotropic graphene framework using this multiscale structural modulation, the graphene skeleton of the higher‐density DAGF has a closer contact between the adjacent graphene sheets with a larger overlapping area of graphene–graphene along the skeleton direction, thus leading to the direct improvement of the intrinsic *κ*_skeleton_ with an increase of the density.

In order to in‐depth study the quantitative relationship between the overlapping area of the adjacent graphene sheets and the thermal conductivity of the graphene skeleton for different DAGFs, a nonlinear model proposed by Foygel et al. was applied to analyze the *κ*_⊥_ of the DAGF1/EP and DAGF5/EP, respectively.^[^ ^64^, ^65^, ^66^ ^]^ As the thermal conductivity model given by Equation (2), the graphene sheets were chosen as the research object, and the contact resistance (*R*_contact_) and overlapping area (*S*) of adjacent graphene sheets can be estimated using the Equations (3) and (4)

(2) $$\kappa_{⊥}-\kappa_{\mathrm{EP}}=\kappa_{0}{\left(\frac{f-f_{c}}{1-f_{c}}\right)}^{\tau }$$

(3) $$R_{\mathrm{contact}}=\frac{1}{\kappa_{0}L{\left(f_{c}\right)}^{\tau }}$$

(4) $$S=\frac{R_{\mathrm{int}}}{R_{\mathrm{contact}}}=R_{\mathrm{int}}\kappa_{0}L{\left(f_{c}\right)}^{\tau }$$

Among Equations ((2), (3), (4)), *κ*_⊥_ is the through‐plane thermal conductivity of the composites versus the volume fraction (*f*); *κ*_EP_ is the thermal conductivity of the epoxy matrix; *κ*_0_ is the pre‐exponential factor ratio related to the contribution of graphene sheets; *f*_c_ is the critical volume fraction of graphene sheets, and *τ* is a conductivity exponent; *L* is the plate size of the graphene sheets (≈5.4 µm); *R*_int_ is the interfacial thermal resistance of the overlapped graphene sheets based on the van der Waals (vdW) interaction, and therefore the *R*_int_ for the two cases is the same, ideally, at the order of magnitude level of 10^−9^ K m^2^ W^−1^.^[^ ^21^ ^]^ Based on the experimental *κ*_⊥_ of DAGF1/EP and DAGF5/EP, as well as the corresponding change trend predicted using the Equation (1), the values of *κ*_0_, *τ*, and *f*_c_ for the two cases can be calculated, as listed in Table S3 (Supporting Information). According to Equation (3), we obtained that the overlapping area of adjacent graphene sheets for the case of DAGF5/EP (9.56 × 10^−14^ m^2^) is ≈2.1 times as high as that of DAGF1/EP (4.57 × 10^−14^ m^2^). Moreover, based on the result of nonequilibrium molecular dynamics (NEMD) simulation (Figure 5g,h; and Figure S9, Supporting Information ), we demonstrate that a 2.1‐times enhancement of the overlapping area for adjacent graphene sheets can improve the junction thermal conductivity along the basal plane direction by ≈310% (DAGF1/EP: 0.089 W m^−1^ K^−1^, DAGF5/EP: 0.365 W m^−1^ K^−1^). The detailed calculation and analysis of the Foygel model and the NEMD simulation can be found in Section S3 and Figure S9 (Supporting Information). And the results confirm the superior *κ*_skeleton_ of an individual graphene skeleton for the higher‐density DAGFs, and provide fundamental evidence to explain the extraordinary *κ*_⊥_ enhancement of DAGF/EP with the increase of the graphene content. As a result, the *κ*_⊥_ enhancement of the corresponding DAGF5/EP can be as high as 325 times that of neat epoxy at the graphene content of 13.3 vol%. To the best of our knowledge, this *κ*_⊥_ enhancement achieved is the highest value ever reported for graphene/polymer composites with a similar level of graphene addition (Figure 5a), and gives a remarkably high specific TCE (TCE per 1 vol% graphene content) over 2400%, significantly outperforming previously reported results.

### 2.3 Solar‐Thermal Energy Conversion Performance of DAGF/Polymer Composites

**Figure 6:**
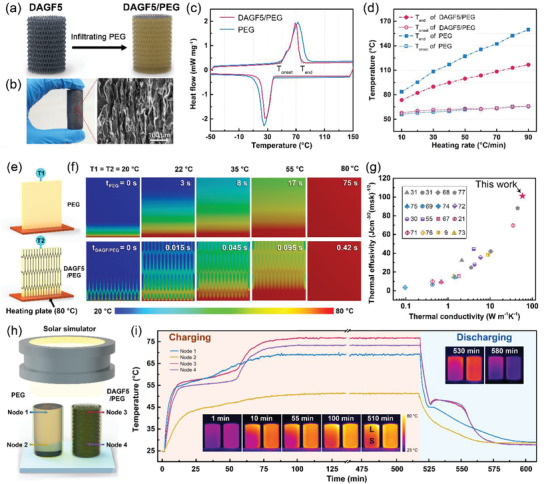
a) Schematic illustrating the fabrication process of the DAGF5/PEG with the corresponding photograph and cross‐sectional SEM image showing in (b). c) DSC heating and cooling scan curves for pure PEG and DAGF5/PEG with a heating rate of 10 °C min^−1^. d) The *T*_onset_ and *T*_end_ of PEG and DAGF5/PEG versus the DSC heating rate. e) Schematic of the ANSYS simulation models and f) the calculated transient temperature distribution for PEG and DAGF5/PEG. The temperature of the heating plate is maintained at 80 °C. g) A comparison of the thermal conductivity and the thermal effusivity of our DAGF5/PEG and with the reported carbon‐based phase‐change composites. h) Schematic illustrating the solar‐thermal energy conversion measurement. i) Temperature evolution curves for PEG and DAGF5/PEG under stimulant solar irradiation. The insets show the infrared images of the two samples during the charging and discharging process.

Efficient thermal energy harvesting using PCMs has enormous potential for cost‐effective energy storage and waste heat recovery.^[^ ^10^ ^]^ However, the low intrinsic thermal conductivity of PCMs (< 0.5 W m^−1^ K^−1^) resulting in a limited speed for the energy conversion is an everlasting bottleneck, which has caused a low efficiency for energy charging/discharging.^[^ ^9^ ^]^ Numerous studies have indicated that the incorporation of PCMs with 3D graphene frameworks is a feasible solution to develop highly thermally conductive PCM‐based composites for solving this problem.^[^ ^31^, ^55^, ^67^ ^]^ Here, based on the excellent performance of our DAGFs in enhancing the thermal conductivity of the polymer matrix, we combined the PEG (a common PCMs, 0.29 W m^−1^ K^−1^) with the as‐prepared DAGF5 using a conventional infiltration method (**Figure** **6**a) and named the resultant composite as DAGF5/PEG. In Figure 6b, the obtained DAGF5/PEG composite with a diameter of 2 cm and a height of 5 cm presents a highly ordered microstructure composed of vertically aligned graphene sheets, in agreement with that of DAGF5/EP, by which the DAGF5/PEG has an ultrahigh *κ*_⊥_ of 58.6 ± 2.2 W m^−1 ^K^−1^ with ≈13.3 vol% graphene addition.

The differential scanning calorimetry (DSC) curves (Figure 6c) indicate that the calculated phase transition enthalpy (Δ*H*_m_) of DAGF5/PEG is 139.7 J g^−1^, which is about 78% that of neat PEG (179.2 J g^−1^), attributing that the additional graphene component did not undergo a phase change. Besides, as shown in Figure 6c,d; and Figure S10 (Supporting Information), the onset melting temperature (*T*_onset_) of DAGF5/PEG is approximately the same as that of neat PEG, when the DSC heating rate was changed from 10 to 90 °C min^−1^, suggesting that the embedding of graphene into PEG does not effect on its normal solid–liquid phase transition. Interestingly, in contrast to the *T*_onset_, the *T*_end_ (end melting temperature) of DAGF5/PEG (73.4 °C) presents a significant decrease of about 10 °C compared to that of PEG (83.6 °C) at the heating rate of 10 °C min^−1^, and this temperature difference (Δ*T*_end_) is further raised to 43 °C, as the heating rate up to 90 °C min^−1^. We proposed that the faster phase‐change speed of DAGF5/PEG compared to that of PEG can be attributed to its superior thermal conductivity (DAGF/PEG: 58.6 W m^−1 ^K^−1^, PEG: 0.2 W m^−1 ^K^−1^), substantially deriving from the highly ordered graphene sheets acted as the continuous heat channels within the PEG matrix.

Accordingly, a finite element modeling using a commercial computational fluid dynamics software (ANSYS) is implemented to simulate the transient thermal response of neat PEG and DAGF5/PEG, respectively, for mimicking the DSC heating process. The simulation model of the two samples was shown in Figure 6e, in which the initial system temperature is 20 °C, and the heating plate is maintained at 80 °C at the bottom side of the two modules, leading to the formation of 1D heat conduction through the PEG and DAGF5/PEG. T1 and T2 are the temperature measurement points located at the top side of the PEG and DAGF5/PEG modules, respectively. More details about the simulations can be seen in Figure S11 (Supporting Information). As the calculated transient temperature distribution shown in Figure 6f, when the top side of the two modules achieves the same temperature rise of 2 °C (T1 = T2 = 22 °C), the thermal response time of PEG and DAGF5/PEG is 3 and 0.015 s, respectively, indicating that the embedding of DAGF5 can increase heat transfer rate by about a factor of 200 compared to that of neat PEG. As a result, in Figure 6f; and Figure S7e,f (Supporting Information), the time needed to reach the equivalence point (T1 = T2 = 80 °C) for the DAGF5/PEG module is only 0.45 s, which is more than two order of magnitude lower than that of neat PEG (75 s). This simulation results directly demonstrate that the high thermal conductivity of DAGF5/PEG is crucial in achieving a faster phase transition speed than that of neat PEG, leading to a significant reduction of phase transition delay time during the DSC heating process. For PCMs, the thermal effusivity ($e=\sqrt{\kappa \rho \Delta H_{m}}$) can be used to evaluate the capability to exchange thermal energy with the surroundings, where *κ*, *ρ*, and Δ*H*_m_ are the thermal conductivity, density, and phase change enthalpy of the PCMs, respectively.^[^ ^68^ ^]^ As shown in Figure 6g; and Table S4 (Supporting Information), based on the ultrafast phase‐change speed originated from the superior thermal conductivity of 58.6 W m^−1 ^K^−1^, our DAGF/PEG exhibit a record‐high thermal effusivity as compared to the currently reported carbon‐based phase‐change composites.^[^ ^9^, ^21^, ^30^, ^31^, ^55^, ^67^, ^68^, ^69^, ^70^, ^71^, ^72^, ^73^, ^74^, ^75^, ^76^, ^77^ ^]^

Given the practical application of thermal energy storage and management, DAGF5/PEG could be employed as a solar‐thermal energy conversion materials, which work by the transformation of the absorbed solar energy at the surface into the thermal energy through a phase transition of the applied PCMs. The solar‐thermal conversion performance test for neat PEG and DAGF5/PEG are illustrated in Figure 6h, in which a xenon lamp was applied as the solar simulator with an intensity of 1.5 sun, and the two samples in quartz crucibles have the same diameter of 2 cm and height of 3 cm. To record the real‐time temperature change and temperature gradient of the test specimen, four thermocouples were inserted into the top and the bottom positions of the two samples (Node 1, 2 for PEG and Node 3, 4 for DAGF5/PEG), respectively, and the total temperature profile evolution was captured using a calibrated infrared camera. More details of the light‐to‐thermal energy conversion measurement can be seen in Figure S12 (Supporting Information). In Figure 6i, when the neat PEG and DAGF5/PEG were solar‐heated from their top surface, the temperature of both increases over time. And in the steady‐state (100–510 min) during the charging process, the recorded temperature at the top and the bottom position of PEG is 69.1 °C (Node 1) and 51.3 °C (Node 2), respectively. It suggests that the PEG cannot completely accomplish a phase change process (liquid/solid ratio, *L*/*S* ≈ 36: 64) after 500 min of illumination, as shown in the IR images in the inset of Figure 6i. This low efficiency of solar energy storage can be attributed to the intrinsic low thermal conductivity of PEG (0.29 W m^−1 ^K^−1^), which prevents the rapid spreading of absorbed heat energy from the top surface into the interior of the sample, showing a large temperature gradient of 8.9 °C cm^−1^ along the vertical direction the sample. In contrast to the neat PEG, after being illuminated for 55 min, the phase transition of the entire DAGF5/PEG can accomplish with a steady‐state temperature of 76.2 °C (Node 3) and 73.4 °C (Node 4) at the top and the bottom position, respectively. And at the steady‐state of the charging process, the temperature gradient through the DAGF5/PEG is only 1.4 °C cm^−1^, demonstrating a much faster heat transfer efficiency, due to the efficient heat propagation along the interconnected graphene skeleton. During the heat discharging process without the light source, the temperature of the DAGF5/PEG is always higher than that of the PEG. Moreover, different from the gradual decrease of the temperature of the PEG, the DAGF5/PEG presents a temperature plateau (46.5–48.5 °C) between 530 and 550 min, suggesting that the heat energy release for DAGF5/PEG can be maintained at a relatively high‐quality level in a wide range of cooling time. Besides, compared to the large temperature difference between the top and bottom of PEG, the temperature gradient through the DAGF/PEG is almost zero during the entire heat discharging process, indicating the superior heat discharging efficiency of DAGF5/PEG compared to that of neat PEG. Not only the excellent light‐to‐thermal energy conversion capability, the DAGF5/PEG also exhibits good shape stability and no leakage of PEG during the during the charging/discharging process (Figure S13, Supporting Information). This result demonstrates the promising possibility to achieve highly efficient thermal energy storages by the incorporation of the thermally conductive DAGFs with the conventional phase change materials.

### 2.4 TIMs Performance of DAGF/Polymer Composites

**Figure 7:**
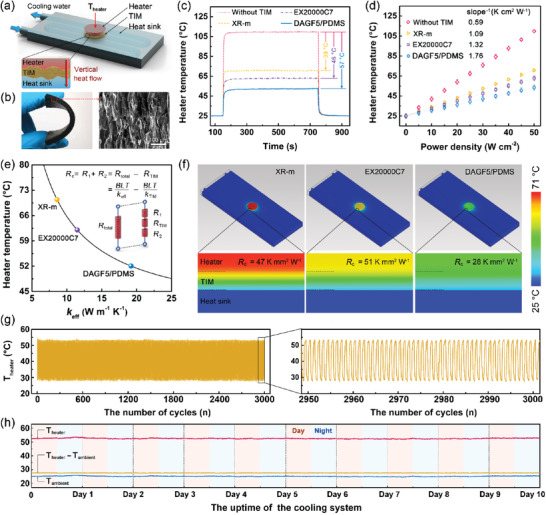
a) Schematic configuration of the TIM performance test system and the heat flow diffusion path along the vertical direction. b) The photograph and the cross‐sectional SEM image of the DAGF5/PDMS composites. The heater temperature evolution versus c) the running time at the power density of 50 W cm^−2^ and d) various power density after heating for 700 s. e) The simulated effective thermal conductivity (*κ*_eff_) of the applied TIM based on the heater temperature shown in (c). f) The comparative heat dissipation capability, according to the simulation results. g) Thermal shock stability in cyclic heating/cooling tests and h) thermal durability in a long‐term TIM performance test (10 days) using DAGF5/PDMS as TIM.

In addition to as thermally conductive fillers incorporated with PCMs for the energy harvesting applications, the excellent capability of our DAGFs in improving the *κ*_⊥_ of the polymer matrix can also endow the composites with considerable potential for use as high‐performance TIMs. TIMs are applied to bridge the heat‐generating electronic components (heater) and the heat sink for dissipating excess heat along the vertical direction, thus expecting to have a high *κ*_⊥_ for maximizing the heat energy transfer efficiency,^[^ ^54^, ^78^ ^]^ as schematically illustrated in **Figure** **7**a. Therefore, we embedded the as‐prepared DAGF5 into PDMS (the commonly used soft matrix of TIMs, 0.18 W m^−1^ K^−1^) and obtain the DAGF5/PDMS composite. For TIM application, the sample was cut into a thin pad with good bendability and compressibility (Figure 7b; and Figure S14, Supporting Information), by which it could sufficiently deform to flatten the asperities on the rough surface under low contact pressure. The cross‐sectional SEM image (Figure 7b) of the composite exhibited a vertically aligned graphene architecture within the PDMS matrix, contributing to the composite with a superior *κ*_⊥_ of 60.2 ± 2.5 W m^−1^ K^−1^ (≈13.3 vol%), which is much higher than that of the state‐of‐the‐art commercial TIMs (10–35 W m^−1^ K^−1^).^[^ ^79^ ^]^

To study the practical cooling performance of the as‐prepared DAGF5/PDMS, a TIM performance test apparatus was built to simulate the actual heat transfer behavior of the electronic devices. In Figure 7a, a circular DAGF5/PDMS with a diameter of 15 mm and a bond line thickness (BLT) of 800 µm was placed between the heater and heat sink at a packaging pressure of 75 psi. For comparison, the identical test conditions for two commercial TIMs with the same size were carried out, including a ceramic particle reinforced thermal pad (≈17 W m^−1^ K^−1^, Fujipoly XR‐m, Japan) and a vertically aligned carbon fiber‐based thermal pad (≈ 35 W m^−1^ K^−1^, Dexerials EX20000C7, Japan). As far as we know, both are state‐of‐the‐art commercial products with the highest thermal conductivity in their respective fields.^[^ ^79^ ^]^ A water cooling system was employed to keep the heat sink temperature constant at 25 °C during the test, and the real‐time temperature evolution of the heater (*T*_heater_) was monitored using a calibrated thermocouple. As the results shown in Figure 7c, compared to the case without TIM, an obvious cooling effect can be found by bridging the heater (50 W cm^−2^) and the heat sink with TIMs. Noticeably, the cooling performance of DAGF5/PDMS with the heater temperature drop of 57 °C is substantially greater than that of XR‐m (39 °C) and EX20000C7 (46 °C) thermal pads. In Figure 7d, according to the linear increase of heater temperature versus the applied power density, the equivalent heat‐transfer coefficients (equal to the reciprocal of the slope^[^ ^80^ ^]^) for the three TIMs can be calculated with the value of 1.76, 1.32, and 1.09 K cm^2^ W^−1^, assigned to the DAGF5/PDMS, EX20000C7, and XR‐m thermal pads, respectively. This result indicates that the system cooling efficiency using DAGF/PDMS as TIM achieves 61% and 33% enhancement compared to that of the XR‐m and EX20000C7 thermal pads, respectively.

A commercial flow solver (IcePak) was then adopted for in‐depth analysis of our test system at a power density of 50 W cm^−2^ (Figure S15a, Supporting Information), and the effective thermal conductivity (*κ*_eff_) of three TIMs was calculated based on the steady‐state heater temperature shown in Figure 7c. As the simulated results shown in Figure 7e; and Figure S15b—d (Supporting Information), the *κ*_eff_ value of DAGF5/PDMS reaches up to 18.6 W m^−1^ K^−1^, which is ≈2.2 times and ≈1.7 times as high as that of XR‐m (8.5 W m^−1^ K^−1^) and EX20000C7 (10.9 W m^−1^ K^−1^) thermal pads, respectively. Besides, based on the equation: *R*_c_ = BLT/*κ*_eff_ − BLT/*κ*_TIM_, our DAGF5/PDMS has a lower thermal contact resistance (two sides) of 28 K mm^2^ W^−1^ compared to that of state‐of‐the‐art commercial thermal pads (XR‐m: 47 K mm^2^ W^−1^, EX20000C7: 51 K mm^2^ W^−1^). The details can be seen in Table S5 (Supporting Information), where the BLT and the *κ*_TIM_ are the thickness in the packaging state and the through‐plane thermal conductivity of the applied TIMs, respectively. The lower contact thermal resistance of our DAGF5/PDMS can be attributed to its lower filler volume fraction (≈13.3 vol%) compared to that of the commercial thermal pads (50–70 vol%), leading to more soft matrix material directly in contact with the rough surface of the heater/heat sink with a better gap‐filling. As a result, combining the dramatically higher through‐plane thermal conductivity and the relatively lower contact thermal resistance, the simulated temperature profiles demonstrate the excellent heat dissipation capability of our DAGF5/PDMS for TIM application, as shown in Figure 7f.

Additionally, a cyclic thermal shock test using DAGF5/PDMS as TIM was carried out by alternatively switching the power density of the heater between 5 and 50 W cm^−2^. The measurement results in Figure 7g indicates a remarkably steady performance in heat dissipation of our DAGF5/PDMS during continuous heating/cooling impact of the device for 3000 times. Figure 7h presents the result of a long‐term TIM performance examination of DAGF5/PDMS by continuously running the test apparatus (Figure 7a) at 50 W cm^−2^ for 10 days in a real environment, and the temperature of the heater (*T*_heater_) and the ambient (*T*_ambient_) were captured using a thermocouple. The *T*_heater_ showed a fluctuated variation over time, attributing to the ever‐changing *T*_ambient_, which was vulnerable to the large temperature difference between day and night, as well as the changes in the weather during the test period. Despite that, the temperature difference between the heater and the ambient (*T*_heater_ – *T*_ambient_) remains almost unchanged during the whole period, indicating an excellent long‐term durability of DAGF5/PDMS as TIM. The comparative TIM performance test suggests that by incorporating DAGF5 with soft PDMS matrix, the obtained composites can be an up‐and‐coming candidate to replace the state‐of‐the‐art commercial thermal pad for dealing with the ever‐increasing thermal energy management issue of next‐generation advanced electronic devices.

## 3 Conclusion

In summary, we developed a highly ordered graphene framework with an intimate contact of its internal adjacent graphene sheets to achieve a superior specific TCE for polymer composites by a dual‐assembly strategy. We demonstrated that the dual‐assembly strategy with a continuous stress‐induced orientation process can not only manipulate the arrangement of the graphene skeleton leading to the formation of highly ordered architecture, but also further reduce the junction thermal resistance between the adjacent graphene sheets by enhancing the overlapping area. As a result, the as‐prepared DAGF5 exhibited superior performance in improving the thermal conductivity of polymers, dramatically enhancing the *κ*_⊥_ of epoxy by ≈325 times (62.4 W m^−1^ K^−1^). To the best of our knowledge, this value achieved is the currently highest value for the graphene framework/polymer composite, and gives an ultrahigh specific TCE over 2400%. Additionally, given the practical applications thermal energy management, the preparation and the performance study of the DAGF5/PEG and DAGF5/PDMS composites were simultaneously reported in this work. We demonstrated that the DAGF5/PEG as PCMs achieved an increased heat transfer rate by ≈200 times that the neat PEG in the solar–thermal energy conversion, and the DAGF5/PDMS performed excellently in application as TIM with the cooling performance‐enhancing by 33–61% compared to that of state‐of‐the‐art commercial TIMs. The present work provides insights into the construction of graphene‐based thermally conductive composites, which may satisfy the thermal energy management requirements arriving from the rapid developments of electronic and energy technologies. Besides, compared to the various techniques ever reported, such as CVD (chemical vapor deposition) growth of graphene framework or CNT (carbon nanotube) sponge, the proposed dual‐assembly strategy, involving a rolling process and high‐temperature annealing, is relatively more straightforward and cost‐effective. It can significantly raise the possibility for the practical application of the as‐prepared thermally conductive graphene‐based composites. Furthermore, this method is not limited to graphene sheets but can also be applied toward the assembly and design of the other 2D nanomaterials (boron nitride nanosheets, MXenes, and etc.) into a macroscopic configuration for more possible practical application.

## 4 Experimental Section

##### Materials

Graphene sheets with the average lateral size of 5.4 µm and thickness of 10.6 nm were prepared through the intercalation and exfoliation of graphite. Porous PU film was obtained from Suzhou Shutao Medical Supplies Co., Ltd. (China). The epoxy matrix (6105) and the hardener (methyl‐hexahydrophthalic anhydride, MHHPA) were purchased from DOW Chemicals (USA) and Shanghai Li Yi Science & Technology Development Co., Ltd. (China), respectively. The Neodymium(III) acetylacetonate trihydrate (Nd(III)acac) was purchased from Aldrich Chemicals. PEG with the numerical‐molecular average weight (Mn) of 4000 was purchased from Aladdin Reagent Co., Ltd. PDMS (Sylgard 184) and the hardener were purchased from Dow Corning Co., Ltd. (Shanghai, China). Ethanol was purchased from Sinopharm Chemical Reagent Co., Ltd. (China). All chemicals were of analytical reagent grade and used without further purification. The Fujipoly XR‐m and the Dexerials EX20000C7 thermal pads were purchased from Fujipoly Trading (Shenzhen) Co., Ltd and Dexerials Corporation (Japan), respectively.

##### Preparation of the DAGF

Graphene/PU monolith was prepared using a dual‐assembly strategy, by which porous PU film was continuously immersed in graphene/ethanol solution (10 mg mL^−1^) to assemble graphene sheets on the surface of PU skeleton, followed by a continuous roll‐to‐roll step to roll up the film into a cylinder (graphene/PU monolith). The film was kept in a tensile state in the roll‐to‐roll process and the stretch ratio was strictly controlled and ranged from 100% to 340% (100%, 160%, 220%, 280%, and 340%). The detailed preparation process of graphene/PU monolith using the stretched PU porous film as the starting template can be found in Figure S4 (Supporting Information). Then, as‐prepared graphene/PU monolith was thermally annealed at 800 °C (1 h) in vacuum for the removal of the PU, followed by the graphitization at 2850 °C (1 h) in an argon atmosphere to obtain a series of DAGF (DAGF1–5).

##### Preparation of the DAGF/EP Composites

Initially, Nd(III)acac was added into epoxy precursor and stirred for 2 h (80 °C) to prepare a homogeneous solution, which was subsequently mixed with a curing agent (MHHPA) at the weight ratio of 100:95 to obtain the epoxy prepolymer. A series of DAGF (DAGF1– 5) was then immersed into the prepolymer for 1 h under vacuum to infiltrate epoxy and remove the air bubbles. Finally, procedural thermal curing of 135 °C (2 h) and 165 °C (14 h) was carried out to obtain the DAGF/EP composites (DAGF1/EP–DAGF5/EP).

##### Preparation of the DAGF5/PEG and DAGF5/PDMS Composites

The DAGF5/PEG composites were fabricated by the infiltration of PEG into porous DAGF5 frameworks with the assistance of a vacuum. The raw PEG powder was first heated to 90 °C to obtain a fully melted PEG with good fluidity. Then, the DAGF5 frameworks were immersed into the melted PEG, and moved the both in a vacuum oven at 90 °C for 5 h to fully infiltrate the PEG and remove the air bubbles. Finally, the samples were cooled at room temperature to obtain the DAGF5/PEG composites. The DAGF5/PEG composites were prepared by the immersion of the DAGF5 frameworks into the mixture of PDMS prepolymer and curing agent with the weight ratio of 50:1. Then the samples were placed in a vacuum oven more than 4 h (room temperature) to remove the air bubbles, followed by curing at 80 °C for 5 h to obtain the DAGF5/PDMS composites.

##### Characterizations

Raman spectra were recorded using a Reflex Raman System (Renishaw plc, Wotton‐under‐edge, UK) employing a laser wavelength of 532 nm. The sample morphologies were examined with field emission scanning electron microscopy (FE‐SEM, Quanta FEG250, FEI, USA). TGA was performed using the TGA 209 F3 (Netzsch, Germany) system to confirm the weight percent of graphene in the polymer matrix. The measurements were carried out under nitrogen in the range from 30 to 800 °C at the heating rate of 10 °C min^−1^. The DSC heating and cooling scan of the PCMs was taken by a PYRIS Diamond system (PerkinElmer, USA). The thermal diffusivities (*α*) of the sample were measured using LFA 467 MicroFlash system (NETZSCH, Germany). The thermal conductivity (*κ*) can be calculated by the equation: *κ* = *α* × *ρ* × *C*_p_, where *ρ* is the measured average density determined by the water displacement method and *C*_p_ is the specific heat capacity of the sample evaluated by using a DSC (PYRIS Diamond, PerkinElmer, USA). The infrared (IR) photos were captured by using an infrared camera (Fluke, Ti400, USA). The compression modulus of DAGF/PDMS was tested using a UTM (model 5567A, Instron, USA). The loading rate was controlled as 0.5 mm min^−1^. The compressive modulus of the samples was obtained by calculating the average value of the tangent modulus (*E* = d*σ*/d*ε*) in the range of 5–30% strain, where *σ* is the compressive stress and *ε* is the corresponding strain. The shape stability of DAGF/PEG was indirectly evaluated by testing the retention rate by using a stress‐controlled rheometer (DHR‐1, TA Instruments, New Castle, DE, USA). During the measurement, the sample was applied a constant pressure of 1 N and heated from 30 to 100 °C (heating rate of 2 °C min^−1^) in a N_2_ atmosphere.

##### Statistical Analysis

Microsoft Excel and OriginPro (version 2015, Originlab Corp.) were used for the statistical analysis of the data presented in this work. All thermal conductivity data were presented as mean ± SD (standard deviation) and the sample size to be tested was listed in Section S1 (Supporting Information). The sample size of the DAGF‐based PCMs and TIMs was detailed descripted in the corresponding Discussion.

## Conflict of Interest

The authors declare no conflict of interest.

## Supporting information

Supporting Information
