# Supplementary material for: Multiscale Structural Modulation of Anisotropic Graphene Framework for Polymer Composites Achieving Highly Efficient Thermal Energy Management
Source: Adv Sci (Weinh). 2021 Feb 19;8(7):2003734. doi: 10.1002/advs.202003734 (PMC8025029; doi:10.1002/advs.202003734)
Supplement: Supplementary file 1 — Supporting Information [file ADVS-8-2003734-s001.pdf]

© 2020 Wiley-VCH GmbH

ADVANCED SCIENCE
Open Access

Supporting Information

for *Adv. Sci.,* DOI: 10.1002/advs.202003734

Multiscale Structural Modulation of Anisotropic Graphene Framework for Polymer Composites Achieving Highly Efficient Thermal Energy Management

Wen Dai, Le Lv, Tengfei Ma, Xiangze Wang, Junfeng Ying, Qingwei Yan, Xue Tan, Jingyao Gao, Chen Xue, Jinhong Yu, Yagang Yao, Qiuping Wei, Rong Sun, Yan Wang, Te-Huan Liu, Tao Chen, Rong Xiang, Nan Jiang, Qunji Xue, Ching-Ping Wong, Shigeo Maruyama and Cheng-Te Lin**

Supporting Information

Multiscale Structural Modulation of Anisotropic Graphene Framework for Polymer Composites Achieving Highly Efficient Thermal Energy Management

Wen Dai, Le Lv, Tengfei Ma, Xiangze Wang, Junfeng Ying, Qingwei Yan, Xue Tan, Jingyao Gao, Chen Xue, Jinhong Yu, Yagang Yao, Qiuping Wei, Rong Sun, Yan Wang, Te-Huan Liu, Tao Chen, Rong Xiang, Nan Jiang, Qunji Xue, Ching-Ping Wong, Shigeo Maruyama and Cheng-Te Lin**

This file includes:

Supplementary Texts

Section S1. Detailed measurement process of the thermal conductivity based laser flash method.

Section S2. The analysis of the thermal conductivity for the DAGF/EP composites using the metal foam theory.

Section S3. The analysis of the thermal conductivity for the DAGF/EP composites according to the *Foygel* nonlinear model.

Supplementary Figures

Figure S1. Raman spectra and XRD patterns of the pristine graphene/PU and DAGF.

Figure S2. Cross-sectional SEM image of the applied porous PU film.

Figure S3. Schematic and TGA analysis of CG/PU and DAG/PU at different sampling regions.

Figure S4. Detailed preparation process of graphene/PU monolith using the stretched PU porous film as the starting template.

Figure S5. The comparison of the generated circumferential stress between in DAGF1/PU and DAGF5/PU.

Figure S6. TGA curves of neat epoxy and DAGF/EP composites in the nitrogen atmosphere.

Figure S7. Photograph of DAGF1 suffering the compression of more than 100 times its own weight.

Figure S8. XRT analysis and high-resolution SEM images the DAGF5/EP composite.

Figure S9. Schematic of the NEMD implementation and the results of the junction thermal conductance for the adjacent graphene sheets with the different overlapping areas.

Figure S10. DSC heating scan curves of pure PEG and DAGF5/PEG with the heating rate changing from 10 to 90 °C min^{-1}.

Figure S11. Schematic of the ANSYS simulation models the results of the transient thermal response for the neat PEG and DAGF5/PEG.

Figure S12. Schematic illustrating the solar-thermal energy conversion measurement of neat PEG and DAGF5/PEG.

Figure S13. The comparison of shape stability between pure PEG and DAGF5/PEG composite.

Figure S14. The comparison of mechanical property between commercial thermal pad and DAGF5/PDMS composite.

Figure S15. Icepak system model and the corresponding results for TIM performance evaluation of DAGF5/PDMS.

Supplementary Tables

Table S1. The parameters for the calculation of in-plane and through-plane thermal conductivity of neat epoxy and the DAGF/EP composites.

Table S2. Comparison of thermal conductivity of our DAGF/polymer composites with reported graphene/polymer composites.

Table S3. The parameters obtained from the Foygel model, and the calculated overlapping area of adjacent graphene sheets.

Table S4. A comparison of the thermal conductivity and the thermal effusivity of our DAGF5/PEG with the reported carbon-based phase-change composites.

Table S5. The detailed parameters for the Icepak simulation implementation.

Table S6. The calculated total thermal resistance and contact thermal resistance of the applied TIMs.

Supplementary Texts

Section S1. Detailed measurement process of the thermal conductivity based laser flash method.

The thermal conductivity of all samples presented was measured by a laser flash method using LFA 467 HyperFlash® system (NETZSCH, Germany). To carry out the measurement, the samples were cut into a square plate with the size of 10 mm × 10 mm × 1 mm. All samples are carefully polished, and coated by a graphite layer (≈ 5 μm) on both upper and lower sides.

In the actual operation, one side of the sample set in the holder was transiently heated by a xenon lamp pulse with the pulse width of 130 ms, and the rise of surface temperature at another side was recorded by an infrared radiation detector. The thermal diffusivity was evaluated by analyzing the fitted curve of temperature evolution versus sampling time based on the equation (S1).

$$\alpha = 0.1388 \frac{d^2}{t_{50}} \qquad \text{(S1)}$$

where α, d, and t_{50} are calculated thermal diffusivity, sample thickness, and the half diffusion time, respectively. The α was obtained by measuring three samples separately for three times, with the average value and standard error shown **Table S1**. Accordingly, the thermal conductivity (κ) could be calculated using equation (S2).

$$\kappa = \alpha \times C_p \times \rho \qquad \text{(S2)}$$

where C_p is the specific heat capacity measured using differential scanning calorimetry (DSC) at 25 °C, and ρ is the density of the samples measured by the Archimedes principle. The C_p and the ρ were obtained by taking the average value of three separate measurements. All the values mentioned above are summarized in **Table S1**.

Section S2. The analysis of the thermal conductivity for the DAGF/EP composites using the metal foam theory.

The thermal conductivity of the composites ($\kappa_{composite}$) embedded with the interconnected graphene framework as a function of the volume fraction (f) can be predicted using the metal foam theory based on the Equation (S3):[S1]

$$\kappa_{composite} = k_{framework} + (1 - f)\,\kappa_{matrix} \qquad \text{(S3)}$$

where f is the volume fraction of the graphene; κ_{matrix} is the bulk thermal conductivity of the polymer matrix; $\kappa_{framework}$ is thermal conductance contributed by the graphene framework (the effective thermal conductivity of graphene framework), and can be calculated according to the Equation (S4):[S2]

$$\kappa_{framework} = \left\langle \cos^2\theta \right\rangle f \kappa_{skeleton} \qquad \text{(S4)}$$

where $\kappa_{skeleton}$ is the solid thermal conductivity of an individual graphene skeleton or graphene strut of the graphene framework; θ is the angle between the graphene skeleton and the direction of heat transfer; the angle bracket indicates the averaging value overall graphene skeleton.

For the case of isotropic graphene framework, the value of the $\left\langle \cos^2\theta \right\rangle$ is taken to be 1/3, indicating that the heat transfer enhancing effect for an isotropic structure as a filler along the three directions (x, y, z-axis) is equal. Therefore, the thermal conductivity of the DAGF1/EP embedded with the quasi-isotropic DAGF1 can be estimated using the Equation (S5):

$$\kappa_{\perp\text{-DAGF1/EP}} \approx \kappa_{//\text{-DAGF1/EP}} = \frac{1}{3} f \kappa_{skeleton} + 1 - f \;\; \kappa_{EP} \qquad \text{(S5)}$$

where $\kappa_{\perp\text{-DAGF1/EP}}$ and $\kappa_{//\text{-DAGF1/EP}}$ are the through-plane and in-plane thermal conductivity of DAGF1/EP measured using the laser flash technique, respectively, and the two are very close, with the average value of ≈ 4 W $m^{-1}K^{-1}$; κ_{EP} is the bulk thermal conductivity of the epoxy matrix (0.19 W $m^{-1}K^{-1}$); f is the volume fraction of DAGF1 in the composite (≈ 2 vol%), based on the TGA analysis. Therefore, the solid thermal conductivity of an individual graphene skeleton ($\kappa_{skeleton}$) for the DAGF1 obtained from Equation (S5) is 560 W $m^{-1}K^{-1}$.

This calculated value was used as the underlying data of the $\kappa_{skeleton}$ for predicted the $\kappa_{\perp}$ of the DAGF/EP composites as a function of the volume fraction.

For the case of anisotropic DAGFx (x = 2 – 5), which is formed by the unidirectional compression of DAGF1, leading to an oriented arrangement of the graphene skeleton along the heat transfer direction, the $\langle \cos^2\theta \rangle$ can be calculated using the Equation (S6) based on the previous reports.[S3] And according to the proportional relationship between the density of DAGFs and the volume fraction of graphene in the DAGF/EP composites (**Figure 4**c and Equation (S7)), the calculation formulas of the $\langle \cos^2\theta \rangle$ for the anisotropic DAGFx (x = 2 – 5) can be converted into Equation (S8).

$$\langle \cos^2\theta \rangle = \frac{1^2}{1^2 + 1^2 + \left(\frac{\rho_{DAGF1}}{\rho}\right)^2} \tag{S6}$$

$$\frac{\rho_{DAGF1}}{f_{DAGF1}} = \frac{\rho}{f} \tag{S7}$$

$$\langle \cos^2\theta \rangle = \frac{1^2}{1^2 + 1^2 + \left(\frac{f_{DAGF1}}{f}\right)^2} = \frac{1}{2 + \left(\frac{f_{DAGF1}}{f}\right)^2} \tag{S8}$$

In Equation (S6 – S8), the ρ_{DAGF1} and f_{DAGF1} were the density and the volume fraction of DAGF1; ρ and f were the density and the volume fraction of the calculated DAGFx (x = 1 – 5). Therefore, based on the Equation (S3 and S4) and Equation (S8), the $\kappa_{\perp}$ of DAGF/EP composites can be predicted by the Equation (S9):

$$\kappa_{\perp\text{-DAGFx/EP}} = \frac{f\kappa_{skeleton}}{2 + \left(\frac{f_{DAGF1}}{f}\right)^2} + 1 - f \ \kappa_{EP} \tag{S9}$$

When the value of $\kappa_{skeleton}$ and κ_{EP} were taken as 560 and 0.19 W m^{-1} K^{-1}, the predicted results of Equation (S9) were plotted and shown in **Figure 5**b (the blue line). The curve can

well fit the first three points (f < 7.2 vol%) of our experimental data, whereas underestimating the $\kappa_{\perp}$ of the last two points. Based on the experimental $\kappa_{\perp}$ of 38.7 and 62.4 W m^{-1} K^{-1} for the DAGF4/EP and DAGF5/EP, respectively, the calculated κ_{skeleton} of DAGF4 and DAGF5 were 770 and 950 W m^{-1} K^{-1} . By taking this value, the predicted $\kappa_{\perp}$ on the basics of Equation (S9) were shown in **Figure 5**b. The yellow line is corresponding to the case of κ_{skeleton} = 770 W m^{-1} K^{-1}, and the red line is the case of κ_{skeleton} = 950 W m^{-1} K^{-1}).

Section S3. The analysis of the thermal conductivity for the DAGF/EP composites according to the Foygel nonlinear model.

The thermal conductivity of the composites with interconnected graphene sheets as filler can be calculated using a nonlinear model proposed by *Foygel* et al., as shown in the following equation:[S4,S5]

$$\kappa_{\perp} - \kappa_{\text{EP}} = \kappa_0 \left(\frac{f - f_c}{1 - f_c} \right)^{\tau} \qquad \text{(S10)}$$

where $\kappa_{\perp}$ is the through-plane thermal conductivity of the composites versus the volume fraction (f) of graphene sheets; κ_{EP} is the bulk thermal conductivity of epoxy matrix; κ_0 a preexponential factor ratio related to the contribution of graphene sheets; f_{c} is the critical volume fraction of graphene sheets and τ is a conductivity exponent.

To solve this nonlinear function, we take a natural logarithm on both sides of the Equation (S10), and obtain Equation (S11):

$$\ln(\kappa_{\perp} - \kappa_{\text{EP}}) = \ln \kappa_0 + \tau \ln(f - f_c) - \tau \ln(1 - f_c) \qquad \text{(S11)}$$

In this equation, $\ln(\kappa_{\perp} - \kappa_{\text{EP}})$ is a function with the volume fraction (f) as an independent variable. Both the $\ln \kappa_0$ and $\tau \ln(1 - f_c)$ are constant terms. Therefore, we can obtain Equation (S12) by taking the derivative on both sides of Equation (S11). Then, the τ can be calculated based on Equation (S13).

$$\frac{1}{\kappa_{\perp} - \kappa_{\mathrm{EP}}} \times \frac{\mathrm{d}\kappa_{\perp}}{\mathrm{d}f} = \frac{\tau}{f - f_c} \qquad \text{(S12)}$$

$$\tau = \frac{f - f_c}{\kappa_{\perp} - \kappa_{\mathrm{EP}}} \times \frac{\mathrm{d}\kappa_{\perp}}{\mathrm{d}f} \qquad \text{(S13)}$$

For the case of DAGF1/EP, the f_c is equal to 0.64 vol% by the tangent process on the experimental data, according to previous reports.[S6] The experimental thermal conductivity data (**Table S1**) indicates that the values of f, $\kappa_{\perp}$, κ_{EP} are 2.03 vol%, 4.07 W m^{-1} K^{-1} and 0.19 W m^{-1} K^{-1}, respectively. Besides, according to the fitting curve shown in **Figure 5**b (the blue line), the $\left.\frac{\mathrm{d}\kappa_{\perp}}{\mathrm{d}f}\right|_{f=2.03} = 311$ can be obtained for the DAGF1/EP composite. Hereby, the τ and κ_0 of the DAGF1/EP calculated from Equation (S13) and Equation (S10) are 1.09 and 417, respectively. By applying the same rules, the f_c, τ and κ_0 for the DAGF5/EP can be calculated, with the values of 0.67 vol%, 0.97 and 464, respectively.

Based on the calculated values of κ_0, f_c and τ for the DAGF1/EP and DAGF5/EP, the contact resistance (R_{contact}) between adjacent graphene sheets for the two cases can be calculated according to the Equation (S14), and the overlapping area (S) of graphene-graphene was estimated using Equation (S15).

$$R_{\mathrm{contact}} = \frac{1}{\kappa_0 L \ f_{\mathrm{c}}^{\ \tau}} \qquad \text{(S14)}$$

$$S = \frac{R_{\mathrm{int}}}{R_{\mathrm{contact}}} \qquad \text{(S15)}$$

where L is the plate size of the graphene sheets (≈ 5.4 μm); R_{int} is the interfacial thermal resistance of the overlapped graphene sheets based on the van der Waals (VdW) interaction, and therefore the R_{int} for the cases of DAGF1/EP and DAGF5/EP is the same, ideally, at the order of magnitude level of 10^{-9} K m^2 W^{-1}.[S7] As a result, according to the Equation (S14–S15), we obtained that the overlapping area of adjacent graphene sheets for the case of

DAGF5/EP (9.56×10^{-14} m^2) is ≈ 2.1 times as high as that of DAGF1/EP (4.57×10^{-14} m^2). The above-mentioned values of can be found in **Table S3**.

Supplementary Figures and Tables

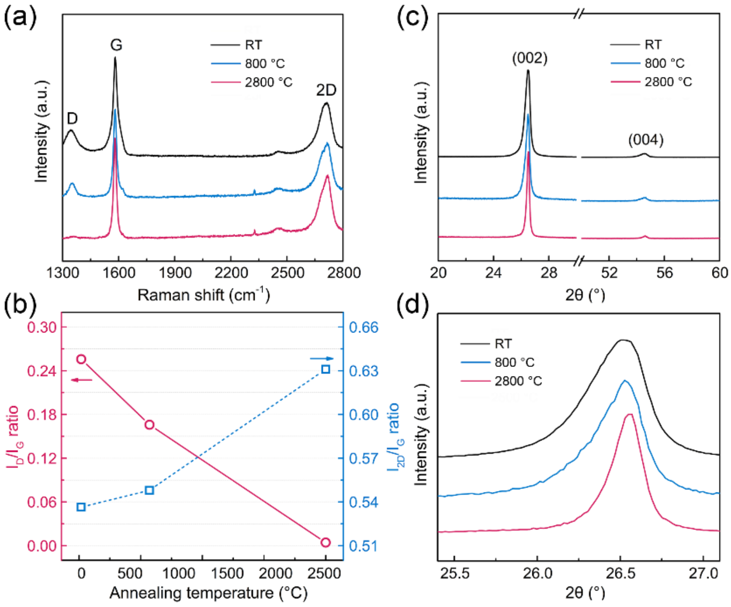

Figure S1. (a) Raman spectra of pristine graphene/PU monolith (black line), DAGF prepared by the pyrolysis of the graphene/PU at 800 °C (blue line), and 2800 °C annealed DAGF (red line). (b) The corresponding changes of I_D/I_G and I_{2D}/I_G ratios from (a). The decrease of I_D/I_G and increase of I_{2D}/I_G ratios indicate the recovery of structural defects of graphene after high temperature treatment.[S8,S9] (c and d) XRD patterns of pristine graphene/PU monolith (black line), DAGF prepared by the pyrolysis of the graphene/PU at 800 °C (blue line), and 2800 °C annealed DAGF (red line). The narrower width of (002) peak suggests and the enlargement of the domain size of graphene along the basal planes after high temperature treatment.[S10,S11]

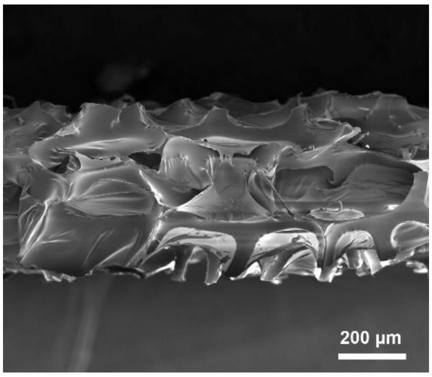

Figure S2. Cross-sectional SEM image of the applied porous PU film, showing a thickness about 500 μm.

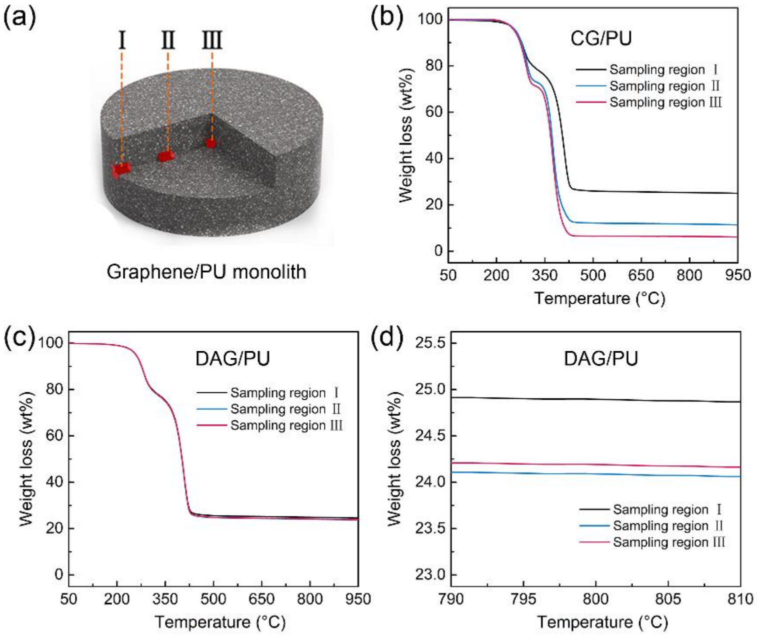

Figure S3. (a) Schematic illustrating three different sampling regions from edge to the central regions within graphene/PU for TGA analysis in the nitrogen atmosphere. TGA curves of (b) CG/PU and (c) DAG/PU at different sampling regions. (d) TGA curves enlarged from (c) at the temperature of 790 – 810 °C, showing an approximate content of graphene sheets (24 – 25 wt%) attaching on the PU skeleton surface.

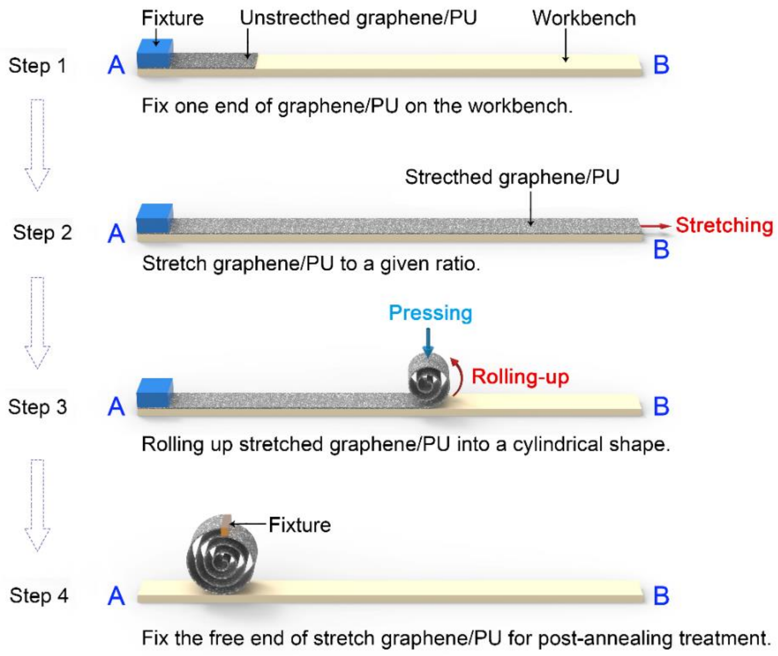

Figure S4. Scheme illustrating the detailed preparation process of graphene/PU monolith using the stretched PU porous film as the starting template.

The detailed preparation of DAGF1–5 using stretched PU film as starting template was presented in **Figure S4**. Firstly, we fixed one end (A-end) of the graphene/PU film (unstretched) to the workbench (**Step 1**), and then stretched the composite film to a given ratio along the workbench (**Step 2**). Finally, we rolled up the stretched graphene/PU film into a cylindrical monolith from the B end of the film (**Step 3**). In this step, a small vertical pressure was applied to the sample to prevent the stretched film from rebounding, and ensure that the film was always attached to the workbench during the rolling process. Accordingly, a constant stretching ratio can be maintained when the film was rolling up. When the rolling process was finished, we fixed the free end of the graphene/PU film on the surface of the cylindrical monolith to prevent the film from unraveling (**Step 4**). Finally, high-temperature annealing of the as-prepared graphene/PU monolith was performed to remove the PU template and obtain the anisotropic graphene frameworks. Based on this experimental process, we can prevent the film from rebounding well, and roll the graphene/PU film into a

cylindrical monolith with a constant stretch ratio. And, the film can always remain in a stretched state, even after it was rolled up and became the units of the graphene/PU monolith.

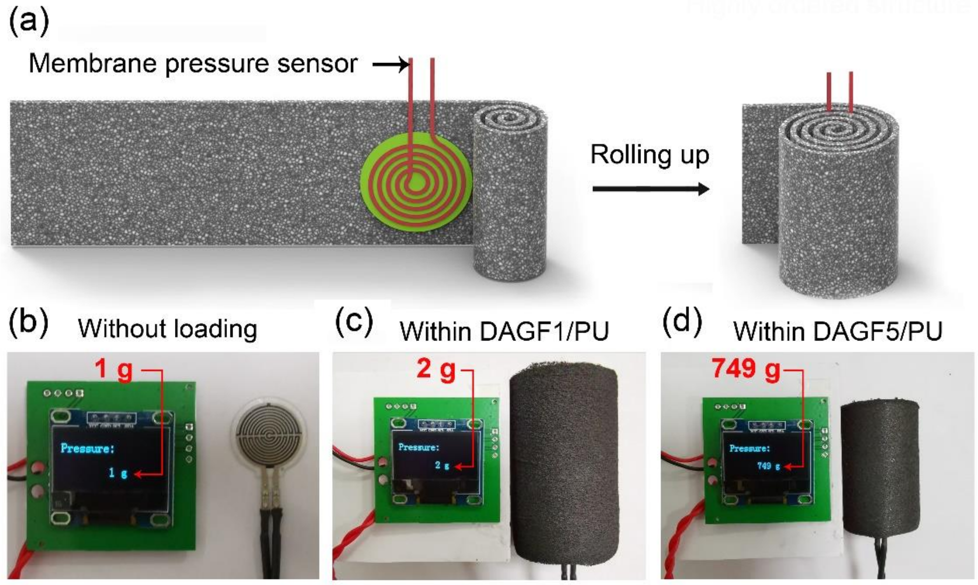

Figure S5. (a) Scheme illustrating the intercalation of membrane pressure sensor into the graphene/PU monolith. (b) Photographs of the pressure sensor without pressure loading, and pressured by the circumferential stress from (c) DAGF1/PU and (d) DAGF5/PU, respectively.

In order to visually demonstrate the circumferential pressure generated by the stretched state of the film, we attached a membrane pressure sensor to the graphene/PU film and rolled them together into a cylinder (**Figure S5a**). As a result, the indication of the pressure sensor within DAGF1/PU monolith (**Figure R5**c) is almost the same as the result without pressure loading (**Figure R5**b), suggesting that there is almost no circumferential pressure in the DAGF1/PU monolith prepared using non-stretched film. In contrast, a much higher pressure can be observed when the pressure sensor was intercalated in DAGF5/PU monolith (**Figure R5**d), confirming the existence of circumferential stress within the monolith prepared using stretched PU film.

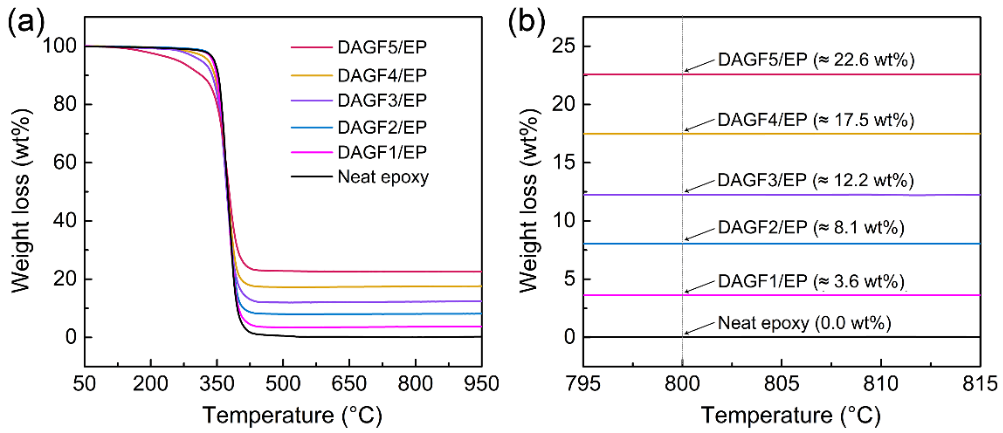

Figure S6. (a) TGA curves of neat epoxy and DAGF/EP composites in the nitrogen atmosphere. (b) TGA curves enlarged from (a) at the temperature of 795 – 815 °C, showing the mass fraction of graphene in the DAGF/EP composites. The volume fraction of graphene (f_{graphene}) can be calculated using the equation of $f_{\text{graphene}} = \frac{\omega_{\text{graphene}} / \rho_{\text{graphene}}}{1 / \rho_{\text{composite}}}$, where ω_{graphene} is the mass fraction of graphene showing in (b), ρ_{graphene} and $\rho_{\text{composite}}$ are the density of graphene (2.1 g cm^{-3}) and the DAGF/EP composite (**Table S1**), respectively.

Table S1. The parameters for the calculation of in-plane and through-plane thermal conductivities of neat epoxy and the DAGF/EP composites. The specific heat capacity (C_p) of all samples was evaluated using a differential scanning calorimeter (DSC) analysis.

	Thermal diffusivity ($mm^2\ s^{-1}$)	Specific heat capacity ($J\ g^{-1}\ K^{-1}$)	Density ($g\ cm^{-3}$)	Thermal conductivity ($W\ m^{-1}\ K^{-1}$)	Direction
Neat epoxy	0.12 ± 0.01	≈ 1.34	≈ 1.189	0.19 ± 0.01	Isotropic
DAGF1/EP	2.54 ± 0.31	≈ 1.32	≈ 1.188	3.98 ± 0.48	$\kappa_{//}$
	2.63 ± 0.24			4.07 ± 0.37	$\kappa_{\perp}$
DAGF2/EP	6.68 ± 1.13	≈ 1.29	≈ 1.208	10.41 ± 1.76	$\kappa_{//}$
	7.18 ± 0.93			11.34 ± 1.45	$\kappa_{\perp}$
DAGF3/EP	9.91 ± 1.43	≈ 1.27	≈ 1.226	15.42 ± 2.23	$\kappa_{//}$
	13.20 ± 1.17			20.55 ± 1.82	$\kappa_{\perp}$
DAGF4/EP	13.18 ± 1.68	≈ 1.24	≈ 1.231	20.12 ± 2.56	$\kappa_{//}$
	25.32 ± 2.08			38.65 ± 3.17	$\kappa_{\perp}$
DAGF5/EP	16.64 ± 1.81	≈ 1.21	≈ 1.234	24.82 ± 2.71	$\kappa_{//}$
	41.81 ± 2.37			62.43 ± 3.54	$\kappa_{\perp}$

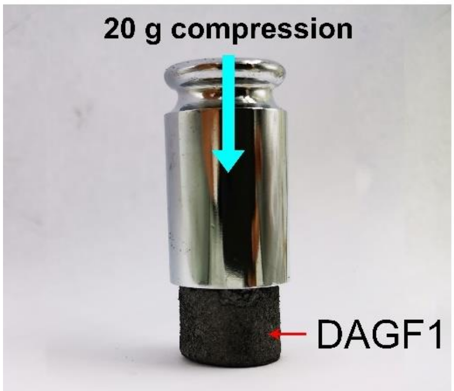

Figure S7. Photograph of DAGF1 suffering the compression of more than 100 times its own weight, indicating that the DAGF framework can have enough strength to undergo the infiltration by epoxy, and maintain its characteristic structure within the epoxy composite.

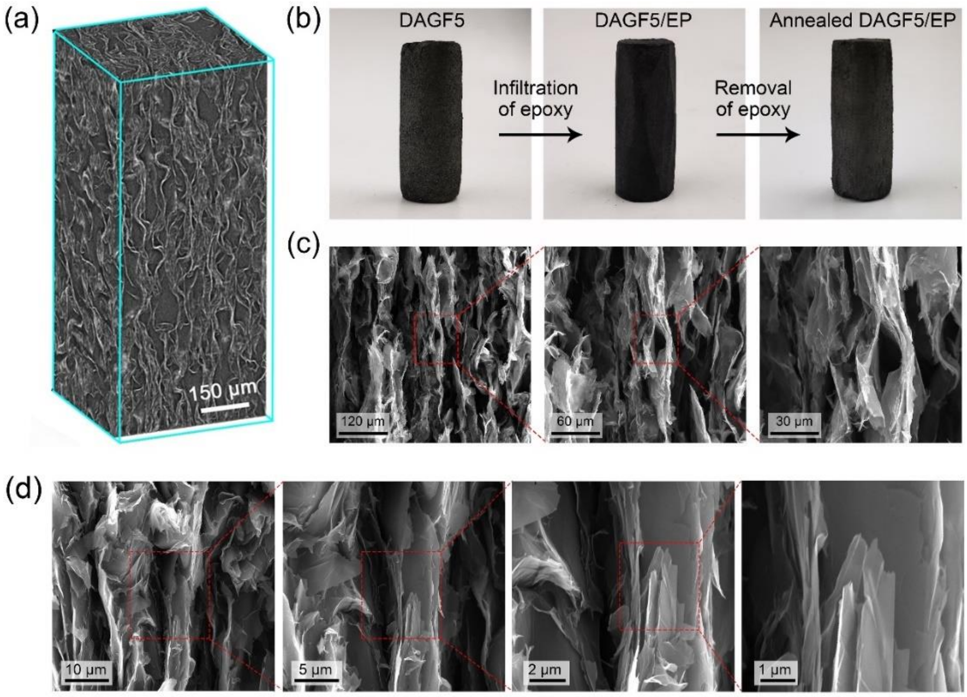

Figure S8. (a) XRT analysis showing the graphene distribution within the DAGF5/EP composite. (b) High temperature annealing of the DAGF5/EP composite for only retaining the graphene framework. (c) Microtopography of the annealed DAGF5/EP. (d) High-resolution SEM images of the DAGF5/EP composite.

X-ray tomography (XRT) technology was performed to analyze the structure of the as-prepared DAGF5/EP composite. In **Figure S8**a, the structure reconstruction based on XRT analysis exhibits an interconnected and anisotropic graphene framework inside the epoxy matrix. Moreover, high-temperature annealing of the DAGF5/EP composite (1,000 °C in the vacuum) was also performed to remove the epoxy for only retaining the graphene framework. As shown in **Figure S8**b, the annealed sample can be free-standing and presents nearly a consistent shape compared to the original DAGF5 framework. In **Figure S8**c, the SEM image of the annealed DAGF5/EP exhibits a densely packed structure composed of highly ordered arrangement of graphene sheets toward the vertical direction. More high-resolution SEM images of DAGF5/EP composite can be found in **Figure S8**d, which clearly exhibits a vertically aligned graphene architecture within the epoxy matrix.

Table S2. Comparison of thermal conductivities of our DAGF/polymer composites with reported graphene/polymer composites.

Filler	Matrix	Thermal conductivity (W m^{-1} K^{-1})	Graphene content (vol%)	TCE (%)	TCE per 1 vol% (%)	Ref.
Multilayer graphene sheets	Epoxy	1.5	2.8	650	232	[S12]
Graphene-SiCNWs foam	Polyimide	2.63	6.54	989	151	[S13]
CVD graphene foam	Paraffin	2.8	1.2	1800	1500	[S1]
CVD graphene foams-CNT	Erythritol	4.1	1.25	411	329	[S14]
Graphite nanoplatelets-CNT	Epoxy	4.6	27.4	2200	80	[S15]
Graphene-multilayer graphene	Epoxy	5.1	10	2300	230	[S16]
Exfoliated graphite	Epoxy	5.8	12.4	2800	226	[S17]
Graphene-Cu nanoparticles	Epoxy	6.9	27.4	3532	129	[S18]
Oriented CVD graphene foam	Epoxy	8.8	4.87	4300	883	[S3]
Graphene framework	Epoxy	10	3.19	5500	1724	[S16]
Vertical graphene nanoflake	PVDF	10	25	5000	200	[S19]
Oriented CVD graphene foam	Rubber	10.64	6.2	8100	1306	[S2]
Graphene-CNT sponge	Polyimide	10.89	2.4	5345	2227	[S20]
Randomly oriented graphene	Epoxy	11	45	5400	120	[S21]
RGO/BN hybrid aerogel	Epoxy	11.01	25.4	5405	213	[S22]
Graphene nanoplatelets	Epoxy	12.4	24.11	6800	282	[S23]
Aligned multilayer graphene	Epoxy	16.75	7.04	8275	1175	[S24]
Vertical graphene framework	Epoxy	35.5	19	17650	929	[S25]
Dual-assembled graphene framework (DAGF5)	Epoxy	62.4	≈ 13.3	32742	2462	This work
	PEG	58.6		20107	1512	
	PDMS	60.2		33160	2593	

Table S3. The parameters obtained from the *Foygel* model, and the calculated overlapping area of adjacent graphene sheets.

	f (vol%)	f_c (vol%)	$\kappa_\perp$ (W m^{-1} K^{-1})	τ	κ_0	$R_{contact}$ (K W^{-1})	S (m^2)
DAGF1/EP	2.03	0.64	4.07	1.09	417.28	1.09E05	4.57E-14
DAGF5/EP	13.3	0.67	62.43	0.97	464.97	5.23E04	9.56E-14

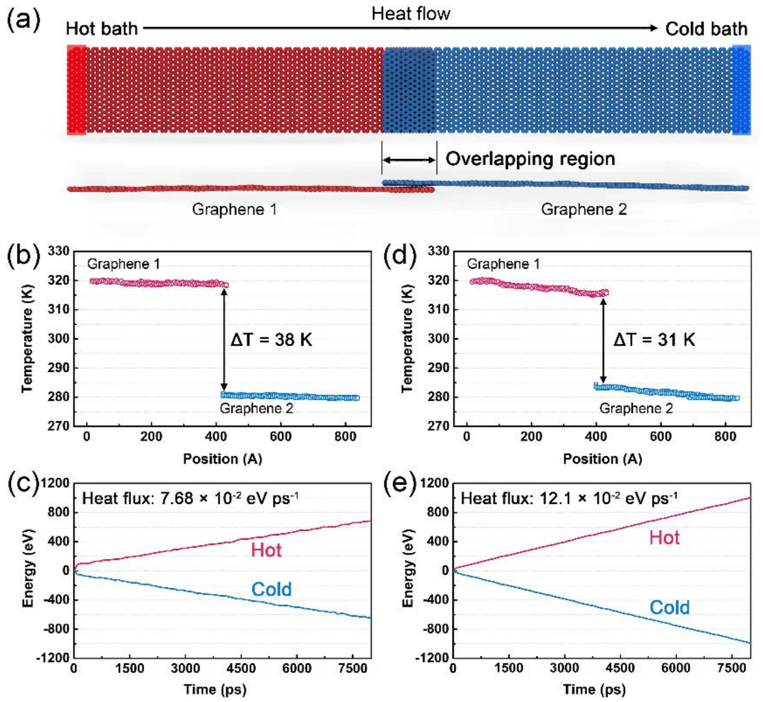

Figure S9. (a) Schematic of the NEMD implementation for the calculation of the junction thermal conductance of the adjacent graphene sheets with different overlapping area. (b–e) The calculated temperature distributions and the heat flux across the two-layer graphene junction structure: (b and c) for the case with the overlapping area of A, and (d and e) for case with the overlapping area of 2.1A.

In order to in-depth study the quantitative relationship between the overlapping area of the adjacent graphene sheets and the thermal conductivity of the graphene skeleton, the non-equilibrium molecular dynamics (NEMD) simulations are performed to calculate thermal transport in the two-layer graphene junction structure, as the simulation model shown in **Figure S9**a. In this implementation, the lateral size of each piece of graphene is 451 Å × 80 Å, and the overlapping regions were set to 15 Å × 80 Å and 32 Å × 80 Å, respectively, for calculating the junction thermal conductance of adjacent graphene with small (A) and large (2.1A) contact area. The LAMMPS molecular dynamics simulator was employed to conduct the NEMD simulations,[26] in which the optimized *Tersoff* potential and the *Lennard-Jones* potential are used to describe intralayer and interlayer C-C interactions, respectively.[S27,S28] Initially, the periodic boundary conditions are applied to all dimensions and random

velocities, with a corresponding average kinetic energy of 5 K, which are assigned to all atoms according to the Gaussian distribution. Subsequently, the relaxation of the structure is applied in the NPT (isobaric-isothermal condition, N: constant number of atoms, P: constant pressure, T: constant temperature) ensemble at 0 Pa and 300 K for 500 ps. After the relaxation process, a few layers of atoms at both ends of the simulation domain are fixed in order to avoid unexpected rotation of the structure and block the heat transfer between heat bathes across the periodic boundary along the length direction. Finally, the atoms in the hot (cold) bath are rescaled to a temperature of 320 K (280 K) in every timestep for a total simulation time of 800 ps, during which the temperature distribution and heat flux of the systems are recorded, as showing in **Figure S9**b–c for the small overlapping area (A) and in **Figure S9**d–e for the large overlapping area (2.1 A). The average temperature drop (ΔT) in the overlapping region is 38 K with an overlapping area of A (**Figure S9**b), slightly higher than 31 K for the case with the overlapping area of 2.1 A (**Figure S9**c). And the heat flux across the two-layer graphene junction structure with the overlapping area of 2.1 A is much larger than that of A case, indicating significantly enhanced thermal conductance of adjacent graphene sheets with high overlapping area. To quantify the effect of the overlapping area of the adjacent graphene and the junction thermal conductance along the basal plane direction, we calculated the junction thermal conductance ($K_{junction}$) using the Equation (S16):

$$K_{junction} = \frac{q}{S_{junction} \times \frac{\Delta T}{L}} \qquad \text{(S16)}$$

where q is the heat current that flows across the system from the hot bath to the cold one; ΔT is the temperature drop between the two sides of overlapping area; L is the overlapping length of two adjacent graphene sheets; $S_{junction}$ is the sectional area of the two-layer graphene junction structure along the heat current direction (6.8 Å × 80 Å). As a result, based on the simulated temperature distribution and heat flux (**Figure S9**b–e), we calculated that the

junction thermal conductivity ($K_{junction}$) along the basal plane direction with the overlapping of 2.1 A (0.365 W m^{-1} K^{-1}) is ≈ 310% higher than that of A case (0.089 W m^{-1} K^{-1}).

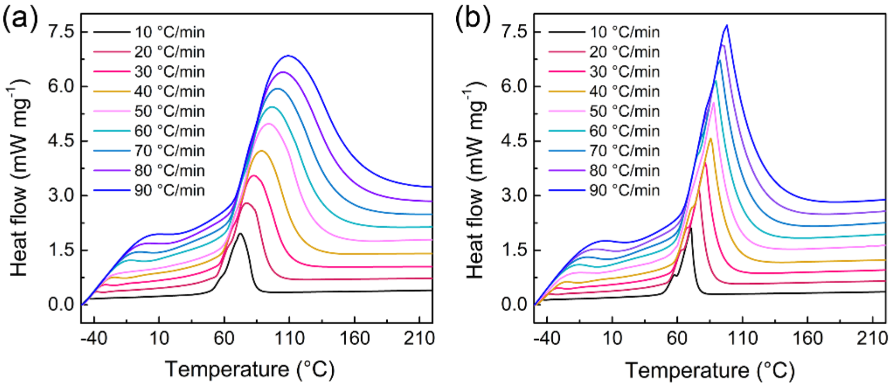

Figure S10. DSC heating scan curves for (a) pure PEG and (b) DAGF5/PEG with the heating rate changing from 10 to 90 °C min^{-1}.

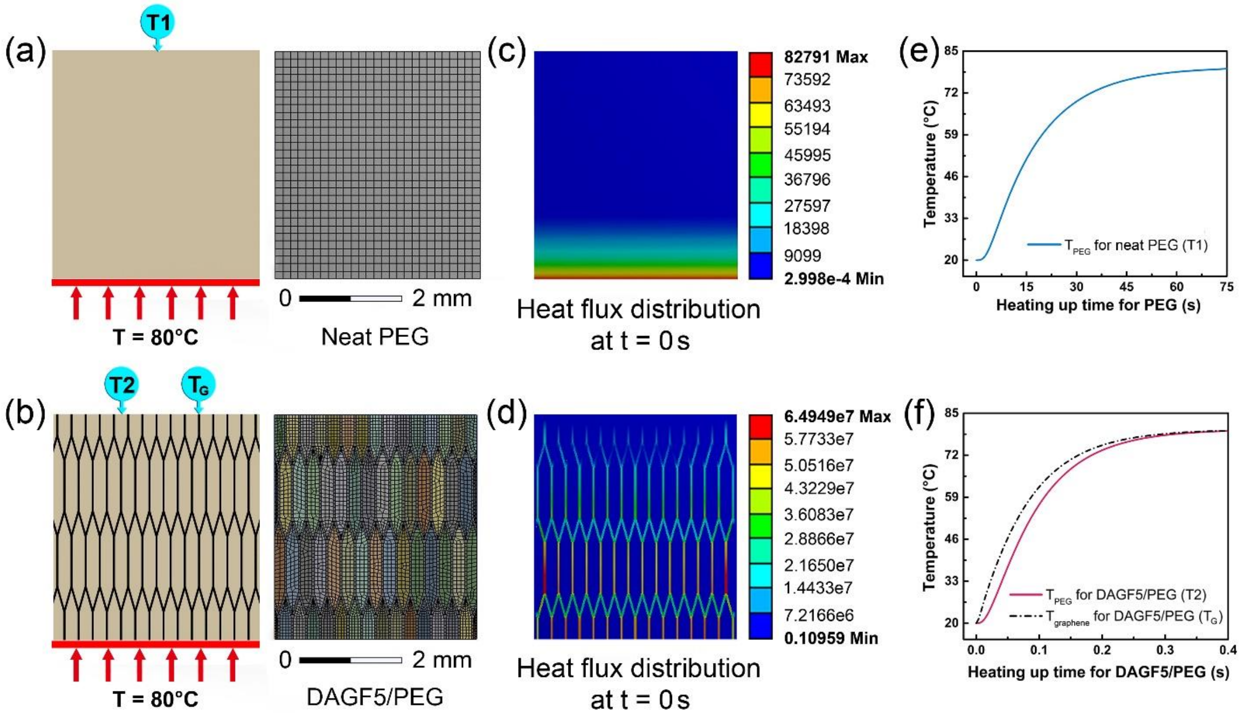

Figure S11. Schematic of the ANSYS simulation models and the corresponding grid division for (a) the neat PEG and (b) DAGF5/PEG modules. Simulated transient heat flux profiles for (c) the neat PEG and (d) DAGF5/PEG modules. The temperature evolution of the measured points versus the heating up time: (e) T1 for the neat PEG and (f) for the DAGF5/PEG

modules.

The finite element (FE) simulations using a commercial computational fluid dynamics software (ANSYS) were performed to analyze the transient thermal response and heat flux distribution of the neat PEG and DAGF5/PEG during heating. As the simulation models shown in **Figure S11**a–b, the computational domain for the two cases is set to 2 mm × 2.2 mm × 0.24 mm. The neat PEG with the thermal conductivity of 0.29 W $m^{-1}K^{-1}$ is shown in brown in **Figure S11**a–b, and the black region in **Figure S11**b refers to the continuous skeleton of the DAGF5 with the volume fraction of ≈ 13.3 vol% and the $\kappa_{skeleton}$ of 950 W $m^{-1}K^{-1}$. The initial temperature of the computational domain is set to 20 °C, and adiabatic boundary conditions are specified at the starting state. T1, T2 and T_G are the three temperature probes located on the top side of the two modulus, thereinto T2 and T_G are located at the PEG area and graphene area on the top side of DAGF5/PEG, respectively, to measure the transient thermal response curves of the two components. When the transient analysis started, the heat source with a constant temperature of 80 °C (schematically illustrated as the red line) was applied at the bottom sides of the two modules, leading to the formation of one-dimensional heat conduction through the PEG and DAGF5/PEG. **Figure S11**c and d present the comparative heat flux distribution of the neat PEG and DAGF5/PEG modulus at the starting point (t = 0 s). The maximal heat flux of the DAGF5/PEG modulus is approximately three orders of magnitude higher than that of neat PEG, and heat flow is mainly distributed along the graphene skeleton, indicating that the DAGF5 severed as the continuous heat channels within the matrices play a key role to achieve a fast thermal response rate. As a result, in **Figure S11**e and f, it just takes 0.45 for the DAGF5/PEG module to reach a steady state (T1 = T2 = 80 °C), whereas the time need for the neat PEG case is as long as 75 s. It can be observed in **Figure S11**f that, the temperature of the graphene component rises faster and always higher as compared to that of PEG until the steady-state, further validating the critical

importance of the DAGF5 in the polymer matrix.

Table S4. A comparison of the thermal conductivity and the thermal effusivity of our DAGF5/PEG with the latest reported carbon-based phase-change composites. Some of the references did not show the density of the phase-change composites. Therefore, the probably maximum thermal effusivity for the composites was calculated by using the maximum density, assuming the carbon-based fillers and the matrix are perfectly mixed without any pores existing inside the composite blocks.

Phase-change composites	Thermal conductivity ($W\ m^{-1}\ K^{-1}$)	Thermal effusivity ($Jcm^{-3/2}(msK)^{-1/2}$)	Ref.
Melamine foam-rGO/paraffin	0.096	3.45	[S29]
Carbon aerogels/paraffin	0.427	6.56	[S30]
3D network carbon/PEG	0.69	9.34	[S31]
Boron nitride nanotube-rGO/PEG	0.43	9.73	[S32]
Graphene framework/paraffin	1.46	14.4	[S33]
Cellulose-graphene aerogel/PEG	1.35	15.4	[S34]
Hybridizing graphene aerogel/paraffin	1.82	15.5	[S35]
Ultrathin-graphite foams/paraffin	3.54	24.7	[S1]
Graphene aerogels/octadecanol	4.28	28.0	[S36]
Ultrathin-graphite foams/erythritol	2.13	32.4	[S1]
Graphene hybrid aerogels/octadecanol	5.92	35.6	[S37]
Anisotropic graphene aerogels/paraffin	8.87	38.3	[S38]
Multi-layer graphene-Cu/octadecane	10.35	42.0	[S39]
Ultrathin-graphite foams-CNT//erythritol	4.1	44.4	[S14]
Oriented graphite sheets/stearic acid (SA)	35	69.7	[S7]
Few-layer graphene sheets/Paraffin	45	88.1	[S40]
DAGF5/PEG	58.6	101	This work

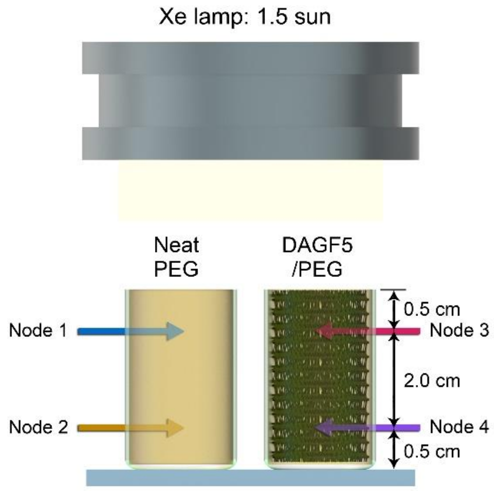

Figure S12. Schematic illustrating the solar-thermal energy conversion measurement.

In this platform, a Xe lamp (HM-Xe500W) equipped with an AM 1.5 G filter was used as the light source to simulate the solar radiation. The neat PEG and the DAGF5/PEG with the same size of Φ 2cm × 3cm were placed into the cylindrical quartz crucibles. When the test was started, the simulated sunlight could irradiate the top surface of two samples with an intensity of 1.5 sun and a radiation time of 520 min, creating continuous heat conduction through the samples along the vertical direction. To monitor the real-time temperature change and temperature gradient across the samples, four thermocouples were inserted into the measured samples. And the position of the four inserted nodes for neat PEG (Node 1, 2) and the DAGF5/PEG (Node 3, 4) was schematically illustrated in **Figure S12**. A multichannel temperature measuring system was used to collect the data of the thermocouples, and the total temperature profile evolution was captured using a calibrated infrared camera (Fluke, Ti400, USA).

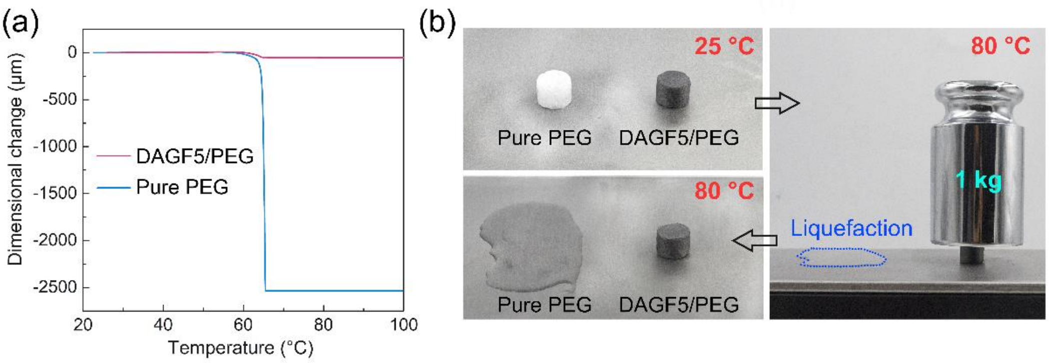

Figure S13. (a) Thermomechanical analyses of pure PEG and DAGF5/PEG composite. (b) Photographs showing the shape stability of pure PEG and DAGF5/PEG composite at 25 °C and 80 °C. The mass of weight is 1 kg.

For the case of phase change composites, in addition to thermal conductivity, the shape stability during phase transition is also essential. Therefore, the shape stability of our DAGF5/PEG is evaluated based on the thermal-mechanical analysis (TMA). As shown in **Figure R13**a, pure PEG melts and cannot keep its shape when the temperature is above 65 °C. On the contrary, the shape of the DAGF5/PEG composite changes little even at 100 °C, attributing to the effective support by the three-dimensional network structure of DAGF5. The shape stability of DAGF5/PEG composite can also be directly demonstrated using digital photos. In **Figure R13**b, in contrast to the direct liquefaction of PEG at 80 °C, the DAGF5/PEG can keep the shape stable without obvious PEG leakage even at a load of 1 kg weight. Combining the high thermal conductivity and good shape stability, our proposed DAGF5/PEG would be very promising for thermal energy storage applications.

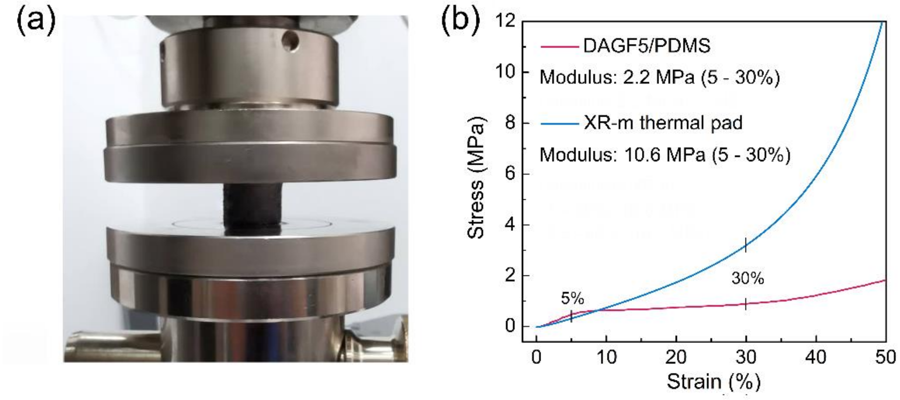

Figure S14. (a) Compression test of XR-m thermal pad and DAGF5/PDMS composite, with the corresponding stress-strain curves showing in (b).

In the actual operation of thermal interface materials (TIMs), a vertical deformation is needed under a packaging pressure (usually less than 1 MPa) for guaranteeing a good contact between the applied TIM and heater/heat sink. Therefore, the compressive property of our DAGF5/PDMS was measured and compared with that of a state-of-the-art commercial TIM (Fujipoly XR-m, Japan). As shown in **Figure S**14, under a normal strain range from 5% to 30%, the compressive stress of DAGF5/PDMS exhibits a change from 0.44 to 0.87 MPa, which falls in the field of normal packaging pressure, conforming to the mechanical property requirements for TIM application. The compressive modulus of the samples was obtained by calculating the average value of the tangent modulus (E = dσ/dε) in the range of 5 – 30% strain, where σ is the compressive stress and ε is the corresponding strain. The calculated results give a lower compressive modulus of DAGF5/PDMS (2.2 MPa) compared to that of XR-m thermal pad (10.6 MPa), indicating an adequate deformation capacity for DAGF5/PDMS as TIM to yield a satisfactory gap-filling.

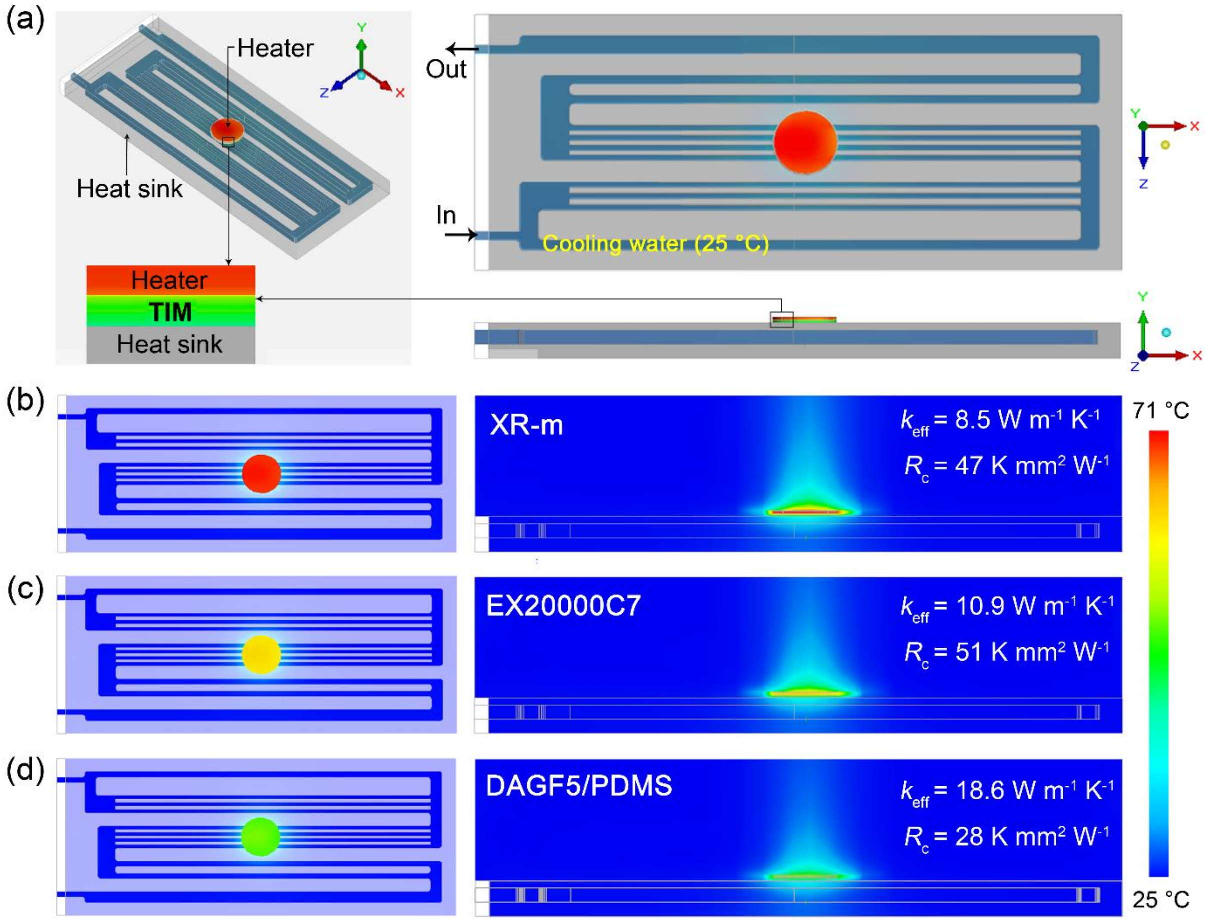

Figure S15. (a) Icepak system model of TIM performance evaluation. The simulated X-Z plane and X-Y plane temperature distribution cloud map of the system with (b) XR-m, (c) EX20000C7 and (d) DAGF5/PDMS as the TIM.

In order to in-depth study the TIM performance in an integrated system using DAGF5/PDMS, a commercial computational fluid dynamics software (Icepak) was employed to simulate the heat transfer process in an electronic system with the power of the device (heater) of 50 W cm^{-2}. The model implementation is presented in **Figure S15**a, and the detailed settings of the heater and the heat sink can be found in **Table S5**. When the simulation was started, the background temperature was set to 25 °C at 1 atm in the atmosphere, and the entrance temperature of cooling water was 25 °C with the volume flow of

400 mL min^{-1}. **Figure S15**b – d shows the steady-state temperature profiles of the simulated system with TIMs, demonstrating a better heat dissipation capability of our DAGF5/PDMS compared to that of XR-m and EX20000C7 thermal pad. In addition, based on the equation: $R_c = R_{total} - R_{TIM} = BLT/\kappa_{eff} - BLT/\kappa_{TIM}$, the total ($R_{total}$) and contact thermal resistances (R_c) of the three TIMs were calculated, with the detailed parameters and results shown in **Table S6**.

Table S5. The detailed parameters of the components in the simulated system.

	Size (cm^3)	Materials	κ (W m^{-1} K^{-1})	C_p (J g^{-1} K^{-1})
Heater	Φ1.5 × 0.1	Alumina	27	0.91
Heat sink	15 × 6 × 0.85	Aluminum alloy	205	0.90

Table S6. The calculated total thermal resistance (R_{total}) and contact thermal resistance (R_c) of the three applied TIMs. The bond line thickness (*BLT*) and the κ_{TIM} are the thickness and the through-plane thermal conductivity of the samples when used as TIMs under packaging conditions. Note that at the packaging pressure of 75 psi, the κ_{TIM} of DAGF5/PDMS is 52.4 W m^{-1} K^{-1}, based on the experimental measurement.

Applied TIMs	*BLT* (μm)	κ_{TIM} (W m^{-1} K^{-1})	κ_{eff} (W m^{-1} K^{-1})	R_{total} (K mm^2 W^{-1})	R_c (K mm^2 W^{-1})
XR-m	800	17.0	8.5	94.1	47.1
EX20000C7	800	35.0	10.9	73.4	50.5
DAGF5/PDMS	800	52.4	18.6	43.0	27.7

Supplementary References

[S1] H. Ji, D. P. Sellan, M. T. Pettes, X. Kong, J. Ji, L. Shi, R. S. Ruoff, *Energy Environ. Sci.* **2014**, 7, 1185.

[S2] Z. Wu, C. Xu, C. Ma, Z. Liu, H. M. Cheng, W. Ren, *Adv. Mater.* **2019**, 31, 1900199.

[S3] X. Shen, Z. Wang, Y. Wu, X. Liu, Y. B. He, Q. Zheng, Q. H. Yang, F. Kang, J.-K. Kim, *Mater. Horiz.* **2018**, 5, 275.

[S4] M. Foygel, R. Morris, D. Anez, S. French, V. Sobolev, *Phys. Rev. B* **2005**, 71, 104201.

[S5] J. Hu, Y. Huang, Y. Yao, G. Pan, J. Sun, X. Zeng, R. Sun, J. B. Xu, B. Song, C.-P. Wong, *ACS Appl. Mater. Interfaces* **2017**, 9, 13544.

[S6] X. Zeng, Y. Yao, Z. Gong, F. Wang, R. Sun, J. Xu, C. P. Wong, *Small* **2015**, 11, 6205.

[S7] S. Wu, T. Li, Z. Tong, J. Chao, T. Zhai, J. Xu, T. Yan, M. Wu, Z. Xu, H. Bao, *Adv. Mater.* **2019**, 31, 1905099.

[S8] L. Peng, Z. Xu, Z. Liu, Y. Guo, P. Li, C. Gao, *Adv. Mater.* **2017**, 29, 1700589.

[S9] L. Cançado, K. Takai, T. Enoki, M. Endo, Y. Kim, H. Mizusaki, A. Jorio, L. Coelho, R. Magalhaes-Paniago, M. Pimenta, *Appl. Phys. Lett.* **2006**, 88, 163106.

[S10] G. Xin, H. Sun, T. Hu, H. R. Fard, X. Sun, N. Koratkar, T. Borca- Tasciuc, J. Lian, *Adv. Mater.* **2014**, 26, 4521.

[S11] C. Teng, D. Xie, J. Wang, Z. Yang, G. Ren, Y. Zhu, *Adv. Funct. Mater.* **2017**, 27, 1700240.

[S12] X. Shen, Z. Wang, Y. Wu, X. Liu, Y. B. He, J. K. Kim, *Nano Lett.* **2016**, 16, 3585.

[S13] W. Dai, J. Yu, Y. Wang, Y. Song, F. E. Alam, K. Nishimura, C. T. Lin, N. Jiang, *J. Mater. Chem. A* **2015**, 3, 4884.

[S14] I. Kholmanov, J. Kim, E. Ou, R. S. Ruoff, L. Shi, A*CS Nano* **2015**, 9, 11699.

[S15] A. Yu, P. Ramesh, X. Sun, E. Bekyarova, M. E. Itkis, R. C. Haddon, *Adv. Mater.* **2008**, 20, 4740.

[S16] H. Hou, W. Dai, Q. Yan, L. Lv, F. E. Alam, M. Yang, Y. Yao, X. Zeng, J. B. Xu, J. Yu, *J. Mater. Chem. A* **2018**, 6, 12091.

[S17] S. Ganguli, A. K. Roy, D. P. Anderson, *Carbon* **2008**, 46, 806.

[S18] Z. Barani, A. Mohammadzadeh, A. Geremew, C. Y. Huang, D. Coleman, L.

Mangolini, F. Kargar, A. A. Balandin, *Adv. Funct. Mater.* **2020**, 30, 1904008.

[S19] H. Jung, S. Yu, N. S. Bae, S. M. Cho, R. H. Kim, S. H. Cho, I. Hwang, B. Jeong, J. S. Ryu, J. Hwang, *ACS Appl. Mater. Interfaces* **2015**, 7, 15256.

[S20] F. Zhang, Y. Feng, M. Qin, L. Gao, Z. Li, F. Zhao, Z. Zhang, F. Lv, W. Feng, *Adv. Funct. Mater.* **2019**, 29, 1901383.

[S21] F. Kargar, Z. Barani, R. Salgado, B. Debnath, J. S. Lewis, E. Aytan, R. K. Lake, A. A. Balandin, *ACS Appl. Mater. Interfaces* **2018**, 10, 37555.

[S22] F. An, X. Li, P. Min, H. Li, Z. Dai, Z. Z. Yu, *Carbon* **2018**, 126, 119.

[S23] M. Shtein, R. Nadiv, M. Buzaglo, K. Kahil, O. Regev, *Chem. Mater.* **2015**, 27, 2100.

[S24] Q. Li, Y. Guo, W. Li, S. Qiu, C. Zhu, X. Wei, M. Chen, C. Liu, S. Liao, Y. Gong, *Chem. Mater.* **2014**, 26, 4459.

[S25] F. An, X. Li, P. Min, P. Liu, Z. G. Jiang, Z. Z. Yu, *ACS Appl. Mater. Interfaces* **2018**, 10, 17383.

[S26] S. Plimpton, *J. Comput. Phys.* **1995**, 117, 1.

[S27] L. Lindsay, D. Broido, *Phys. Rev. B* **2010**, 81, 205441.

[S28] L. Girifalco, M. Hodak, R. S. Lee, *Phys. Rev. B* **2000**, 62, 13104.

[S29] H. Yy. Wu, S. T. Li, Y. W. Shao, X. Z. Jin, X. D. Qi, J. H. Yang, Z. W. Zhou, Y. Wang, *Chem. Eng. J.* **2020**, 379, 122373.

[S30] Y. Wei, J. Li, F. Sun, J. Wu, L. Zhao, *Green Chem.* **2018**, 20, 1858.

[S31] X. Chen, H. Gao, M. Yang, W. Dong, X. Huang, A. Li, C. Dong, G. Wang, *Nano Energy* **2018**, 49, 86.

[S32] M. Wang, T. Zhang, D. Mao, Y. Yao, X. Zeng, L. Ren, Q. Cai, S. Mateti, L. H. Li, X. Zeng, *ACS Nano* **2019**, 13, 7402.

[S33] F. Xue, Y. Lu, X. D. Qi, J.-h. Yang, Y. Wang, *Chem. Eng. J.* **2019**, 365, 20.

[S34] J. Yang, E. Zhang, X. Li, Y. Zhang, J. Qu, Z. Z. Yu, *Carbon* **2016**, 98, 50.

[S35] J. Yang, G. Q. Qi, R. Y. Bao, K. Yi, M. Li, L. Peng, Z. Cai, M. B. Yang, D. Wei, W.

Yang, *Energy Storage Mater.* **2018**, 13, 88.

[S36] J. Yang, X. Li, S. Han, R. Yang, P. Min, Z. Z. Yu, *J. Mater. Chem. A* **2018**, 6, 5880.

[S37] J. Yang, X. Li, S. Han, Y. Zhang, P. Min, N. Koratkar, Z. Z. Yu, *J. Mater. Chem. A* **2016**, 4, 18067.

[S38] P. Min, J. Liu, X. Li, F. An, P. Liu, Y. Shen, N. Koratkar, Z. Z. Yu, *Adv. Funct. Mater.* **2018**, 28, 1805365.

[S39] A. L. Cottrill, A. T. Liu, Y. Kunai, V. B. Koman, A. Kaplan, S. G. Mahajan, P. Liu, A. R. Toland, M. S. Strano, *Nat. Commun.* **2018**, 9, 1.

[S40] P. Goli, S. Legedza, A. Dhar, R. Salgado, J. Renteria, A. A. Balandin, *J. Power Sources* **2014**, 248, 37.
